# Multiphoton Absorption in Branched Quadrupolar Diquinoxalines for Two-Photon Polymerization Design

**DOI:** 10.1021/acsphyschemau.6c00035

**Published:** 2026-05-29

**Authors:** João V. P. Valverde, Welisson de P. Silva, Daniel L. Silva, Nícolas O. Decarli, Mieczysław Łapkowski, Leonardo De Boni, Cleber R. Mendonça

**Affiliations:** † São Carlos Institute of Physics, University of São Paulo, CP 369, 13560-970 São Carlos, SP, Brazil; ‡ Faculty of Chemistry, 366466 Silesian University of Technology, M. Strzody 9, 44-100 Gliwice, GLW, Poland; § Department of Natural Sciences, Mathematics and Education, Federal University of São Carlos, Rod. Anhanguera − Km 174, 13600-970 Araras, SP, Brazil

**Keywords:** Two-photon photoinitiators, two-photon polymerization, two-photon absorption, three-photon absorption, diquinoxaline derivatives, quadrupolar molecules, branched structures

## Abstract

We investigated the photophysical properties of three branched diquinoxaline (**DQ**) derivatives and their potential application as photoinitiators (PIs) for two-photon polymerization (2PP). These compounds exhibit a quasi-centrosymmetric quadrupolar configuration, functionalized with different electron-donating (ED) moieties. We performed detailed linear spectroscopic characterization, as well as the determination of two- and three-photon absorption cross sections (σ_2*PA*_ and σ_3*PA*_), using the spectrally resolved multiphoton-excited fluorescence technique. We complemented the study by performing quantum chemical calculations at the (TD-)­DFT level to obtain relevant information about the electronic structure of the compounds and to model their spectroscopic data. The results showed that the compounds exhibit broad one-photon absorption in the UV–vis region and fluorescence emission at *ca*. 543 nm. Due to their quadrupolar nature, all molecules exhibit significant intramolecular charge transfer. Despite their essentially quadrupolar nature, we observed a symmetry-breaking effect in the relaxed excited state, conferring a dipolar character to the molecules. We observed significant σ_2*PA*_, exhibiting the values up to 230 GM, which was attributed to the enhanced structural planarity, facilitating electron delocalization across all ED branches and resulting in a more efficient additive effect on 2PA response. Additionally, we noted a σ_3*PA*_ of approximately 0.02 × 10^−78^ cm^6^ s^2^ photon^–2^ at 1100 nm. Finally, we confirmed the ability of the **DQ** derivatives to act as two-photon PIs, as demonstrated by the fabrication of 3D microstructures with inherent emissive properties.

## Graphical abstract

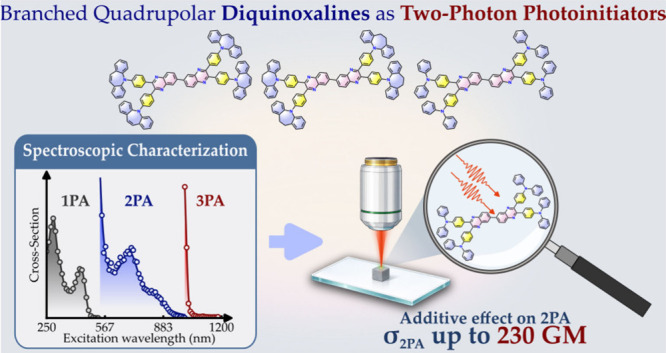

## 1 Introduction

In recent years, two-photon polymerization (2PP) has established itself as one of the leading approaches for micro- and nanoscale 3D printing, offering sub-150 nm spatial resolution.^1–4^ This feature arises from the nature of the two-photon absorption (2PA) process, which, when combined with excitation at energies below one-photon absorption (sub-1PA) and the inherently low transition probability, results in a high penetration depth and spatial confinement.^5,6^ Compared to UV-induced polymerization, 2PP not only offers subdiffraction-limit resolution but also minimizes undesirable effects, such as photochemical side reactions and thermal degradation.^7,8^ Since the demonstration of the first spiral microstructure by Maruo et al.^9^ in the 1990s, the technique has been widely explored across various fields, including micro-optics,^10,11^ microfluidics,^12^ metamaterials,^13^ and biomimetic devices.^14,15^ 2PP may also play a relevant role in enabling the fabrication of micro- and nanodevices for Industry 4.0, such as sensors and actuators, required for real-time monitoring, automation, and adaptive operation.^16–18^

Two-photon polymerization has advanced significantly, leading to the development of specialized commercial platforms including Microlight3D, Femtika, and Nanoscribe. However, this technique still faces challenges that must be overcome to make it scalable, particularly regarding photoinitiators (PIs). Since the early stages, studies have employed PIs originally developed for UV excitation due to their wide commercial availability.^7,8,19,20^ The main limitation of these PIs is their low 2PA cross-section (σ_2*PA*_), typically less than 40 GM at maximum absorption wavelength.^21–23^ In the region around 800 nm, corresponding to the central wavelength of Ti:sapphire lasers widely used in 2PP, these values are even lower (<10 GM).^21–23^ As a consequence, the use of high excitation intensities and prolonged exposure times becomes necessary.^20,21,23^ This hinders the fabrication of high-resolution structures, as the slow polymerization rate can compromise the integrity of the final microstructure. In this context, one of the main challenges lies in developing organic compounds that simultaneously combine a high σ_2*PA*_, efficient performance as PIs, and the ability to generate optically active microstructures. Such properties enable, for example, the creation of fluorescent structures for applications in photonic components, such as high-resolution displays, 3D data storage, anticounterfeiting systems, and tunable-emission devices.^24–26^

Several molecular design strategies have been proposed for the development of organic compounds with high σ_2*PA*_. A common strategy involves the functionalization of a molecule with different combinations of electron-donating (ED) and electron-withdrawing (EW) groups connected through a π-conjugated bridge.^5,27,28^ This approach enables the design of asymmetric dipolar molecules, of the type ED−π–EW, and symmetric quadrupolar molecules, such as ED−π–EW−π–ED or EW−π–ED−π–EW.^5,27^ The quadrupolar design can be up to four times more efficient than dipolar ones, in addition to allowing the incorporation of multiple ED or EW groups, which form branched compounds.^5,27^ These compounds can exhibit two distinct behaviors: (i) an additive effect, in which the branches act independently, and the overall 2PA is the sum of individual contributions; (ii) a cooperative effect, where electronic coupling between the branches leads to an overall 2PA that can exceed the sum.^27,29^ Although branched compounds represent a more refined approach, there are still few studies investigating the influence of different peripheral groups and their performance as two-photon PIs.

In this context, quinoxalines (particularly their derivatives) are considered a promising class of materials due to their favorable properties, including accessible synthesis routes,^30,31^ a high degree of conjugation,^32^ good emissive characteristics,^33–35^ and a strong EW capability.^31,36^ In this scenario, we designed and employed three branched quinoxaline-based molecules with a quasi-centrosymmetric quadrupolar configuration of the type ED_2_–π–EW–EW−π–ED_2_ as two-photon PIs. As illustrated in Figure 1, the molecules feature a central core composed of a binary quinoxaline system (diquinoxaline, **DQ**), which acts as a strong EW, to which different ED branches are connected: 5H-dibenzo­[b,f]­azepine (**IMD**), tetrahydro-3H-dibenzo­[b,f]­azepine (**DDA**), and diphenylamine (**DPA**). Each ED branch exhibits a distinct degree of structural rigidity, which may influence the general molecular conformation and, consequently, its photophysical properties. We provided the details of the synthesis procedures in the Supporting Information (SI).

**Figure 1:**
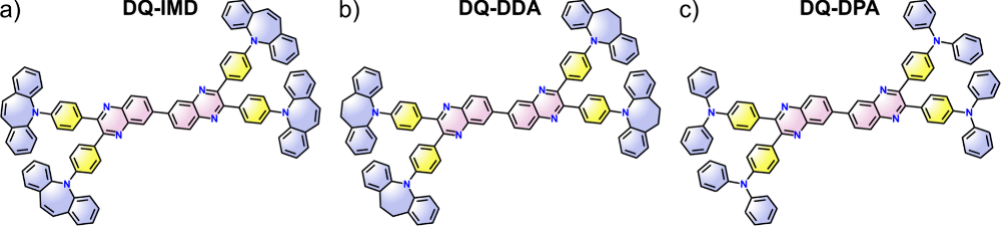
Chemical structure of the diquinoxaline derivatives, named as a) **DQ-IMD**, b) **DQ-DDA**, and c) **DQ-DPA**. The light blue, yellow, and red shaded areas represent the electron-donating branches, the π-conjugated bridge, and the electron-withdrawing groups, respectively.

Here, we investigate the photophysical properties of **DQ** derivatives (**DQ-IMD**, **DQ-DDA**, and **DQ-DPA**), focusing on how structural modifications promoted by the different ED branches influence their behavior. To this end, we performed a series of linear spectroscopic measurements and characterized their 2- and 3PA spectra using the multiphoton-excited fluorescence (MPEF) technique. In addition, we performed quantum chemical calculations (QCC) at the (TD-)­DFT levels to obtain relevant information about the electronic structure of the compounds and to model our experimental spectroscopic data. Finally, we assessed the potential of these compounds as two-photon PIs through the fabrication of microstructures.

## 2 Results and Discussion

### 2.1 One-Photon Absorption and Emissive Properties

Figure 2a-c (black lines) shows the 1PA spectra of the **DQ** derivatives in dichloromethane (DCM). All compounds exhibit two broad absorption bands in the UV–vis spectral region, ranging from 250 to 510 nm (4.96 to 2.43 eV). The lower- energy band is located at *ca*. 434 nm (2.86 eV) with a molar absorptivity of approximately 5.35 × 10^4^ M^–1^cm^–1^ for **DQ-DPA** and **DQ-IMD**, and 9.70 × 10^4^ M^–1^cm^–1^ for **DQ-DDA**. On the other hand, the higher-energy band is situated at *ca*. 289 nm (4.29 eV) with a molar absorptivity of around 9.60 × 10^4^ M^–1^cm^–1^ for **DQ-DPA** and **DQ-IMD**, and 5.50 × 10^4^ M^–1^cm^–1^ for **DQ-DDA**. These high molar absorptivity values suggest that the electronic transitions are of π-π* nature, involving an intramolecular charge transfer (ICT) process (shown further in Figure 3). By analyzing the three 1PA spectra, we can observe that replacing both **IMD** and **DDA** with **DPA** units results in a redshift of approximately 14 nm (0.2 eV) and 26 nm (0.3 eV) of the absorption bands, respectively. This behavior is likely associated with the greater rotational flexibility of the **DPA** moiety, which allows the molecular structure to adopt a more planar conformation (limited by steric hindrance) and, consequently, a more effective electronic conjugation compared to the other molecules. Further analysis of this effect is provided in the following sections.

**Figure 2:**
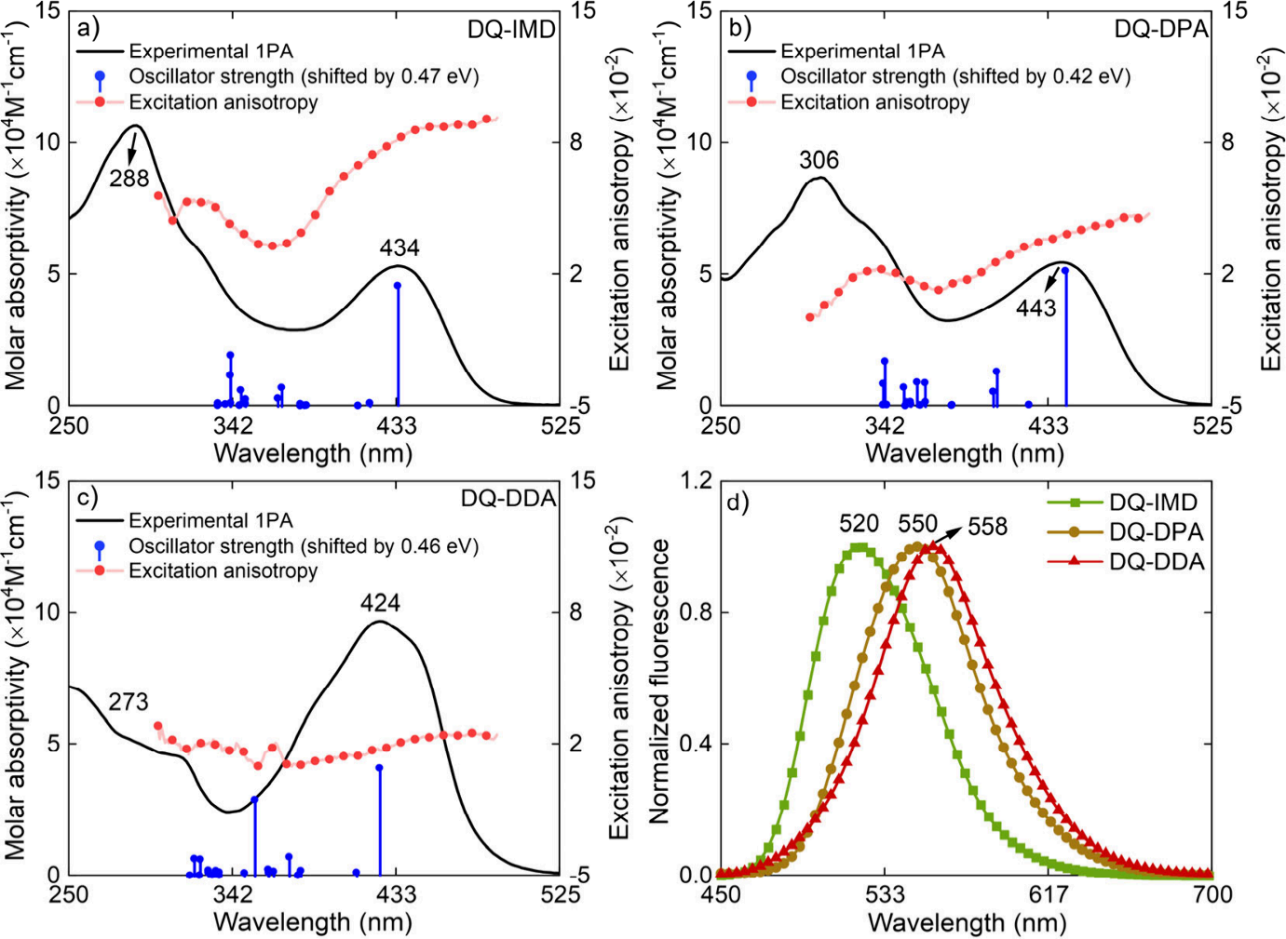
a–c) One-photon absorption (black lines: left axis and bottom axis) and excitation anisotropy spectra (red dots: right axis and bottom axis) of the diquinoxaline derivatives in DCM. The blue vertical sticks with dots at the top represent the oscillator strengths obtained from IEFPCM-TD-CAM-B3LYP/6-311++G­(d,p) calculations (scale from 0 to 7.0). d) Normalized fluorescence emission spectra of all samples in DCM.

**Figure 3:**
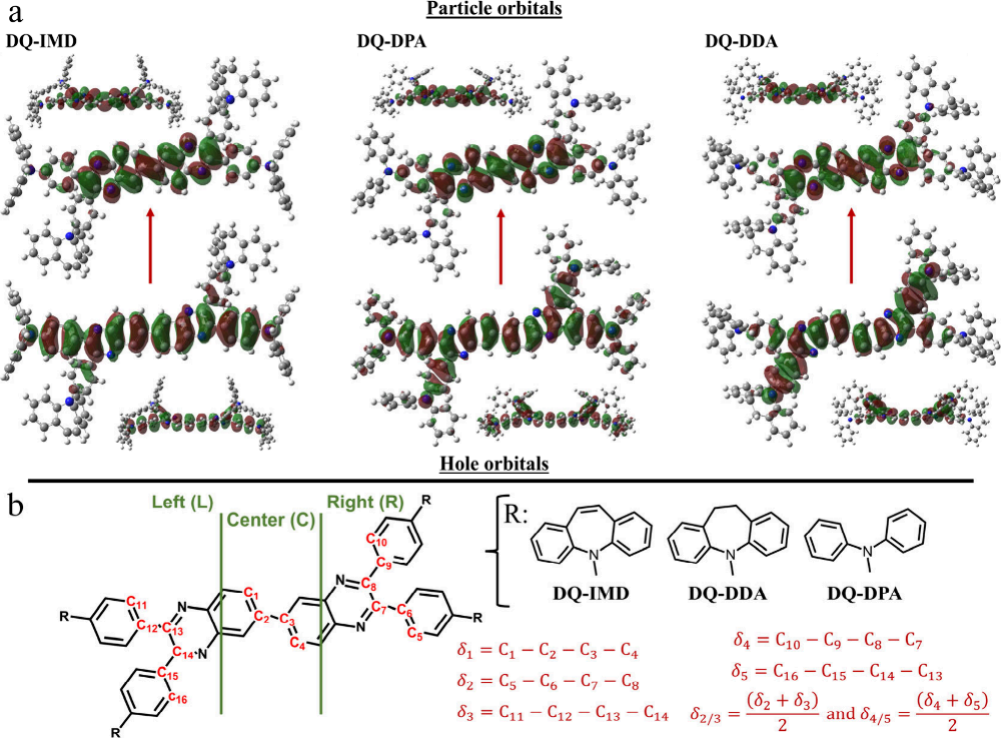
a) Natural transition orbitals corresponding to the *S*_0_→*S*_1_ transition of the diquinoxaline derivatives, obtained from quantum chemical calculations at the IEFPCM-TD-CAM-B3LYP/6-311++G­(d,p) level (isocontour value: 0.015 au). The central images show the front view of the hole (bottom) and particle (top) orbitals, while the insets present the corresponding side views. b) Representation of the central structure of diquinoxaline, highlighting the definition of the dihedral angles δ_1_, δ_2_, δ_3_, δ_4_, δ_5_, δ_2/3_, and δ_4/5_.

The excitation anisotropy spectra (Figure 2a-c, red circles) exhibit slightly different behaviors for the **DQ** derivatives. Upon analyzing the lower-energy band, we noted that, for **DQ-IMD**, the anisotropy remains constant between 434 and 500 nm but then slopes sharply toward wavelengths shorter than 434 nm. For **DQ-DPA** and **DQ-DDA**, the anisotropy shows a continuous slope along the entire band (between 400 and 500 nm). Conversely, in the higher-energy band, the spectra show a more complex form for all samples. This type of behavior, observed in the anisotropy spectra as a whole, occurs when there are energetically close electronic transitions, indicating that both absorption bands are composed of multiple electronic states (*vide infra*).^37^ Furthermore, the anisotropy displays low values even in a high-viscosity medium (average value of 0.02). This effect arises from a substantial conformational change, facilitated by the structural flexibility of **DQs**, and an electronic reorganization in the excited state, which alters the relative orientation between the absorption and emission transition dipole moments (μ_01_ and μ_10_).^38–41^ This process is analyzed in more detail later.

The results of the QCCs at the IEFPCM-TD-CAM-B3LYP/6-311++G­(d,p) level (Figure 2a-c, blue vertical lines, and Table SI4) show that, indeed, multiple electronic transitions with different intensities exist within the two absorption bands, corroborating the observation of the anisotropy spectra. In the lower-energy band, we identify the *S*_0_ → *S*_1_ transition as the most intense, accompanied by transitions with relatively low oscillator strengths (*S*_0_ → *S*_2_, *S*_0_ → *S*_3_, and *S*_0_ → *S*_4_), with values around 0.03, indicating weak accessibility of these states via 1PA. However, as we will see, these states can play a significant role in 2PA. It is important to mention here that to facilitate the comparison between the general trend predicted by the theoretical results and the experimental data, we applied a uniform energy redshift of ∼ 0.45 eV to the theoretical results in Figure 2a–c**)**. The general overestimation of the transition energies is a well-documented behavior of TD-CAM-B3LYP calculations;^42–45^ we provided details of the procedure adopted in Section SI5.

Figure 2d displays the fluorescence emission spectra of the **DQ** derivatives in DCM, while Table 1 summarizes key data on their emissive properties. The fluorescence spectra of all three samples remain independent of the excitation wavelength,^46^ as expected from Kasha’s rule (see Figure SI1). The fluorescence emission occurs in the visible region, peaking at 520 nm for **DQ-IMD**, 550 nm for **DQ-DPA** and 558 nm for **DQ-DDA** (green emission), with a considerable Stokes shift (Δν̅), following the order: **DQ-DDA** > **DQ-DPA** > **DQ-IMD**. This behavior contrasts with that observed for 1PA, suggesting that **DQ-DDA** undergoes greater relaxation in the excited state than the others (*vide infra*).

**Table 1:** Photophysical Properties of the Diquinoxaline Derivatives: Stokes Shift (Δν̅), Fluorescence Quantum Yield (ϕ_fl_), Fluorescence Lifetime (τ_fl_), Radiative Decay Rate (*k*_r_), and Nonradiative Decay Rate (*k*_nr_)

Samples	Δν̅ (cm^–1^)	ϕ_fl_ (%)	τ_fl_ (ns)	*k*_*r*_ (×10^7^ s^–1^)	*k*_nr_ (×10^7^ s^–1^)
**DQ-IMD**	3811 (86 nm)	42 ± 6	2.7 ± 0.3	16 ± 3	21 ± 5
**DQ-DPA**	4391 (107 nm)	21 ± 3	5.7 ± 0.6	3.7 ± 0.7	14 ± 2
**DQ-DDA**	5664 (134 nm)	3 ± 0.5	8.2 ± 0.8	0.37 ± 0.07	11 ± 1

The analysis of the emissive properties (see Table 1) shows that ϕ_fl_ ranges from 3% for **DQ-DDA** to 42% for **DQ-IMD**. In contrast, τ_fl_ varies from 2.7 ns for **DQ-IMD** to 8.2 ns for **DQ-DDA**. By investigating the deactivation pathways, the nonradiative decay rate (*k*_*nr*_) is, on average, 12 times higher than the radiative decay rate (*k*_*r*_). This behavior can be attributed to the intramolecular rotation of the ED branches around the single bonds connecting them to the **DQ** core, significantly contributing to the nonradiative deactivation of the excited state.^47,48^ Furthermore, the smaller difference in decay rates observed for **DQ-IMD** may be associated with the greater rigidity of its peripheral units (**IMD**), which favors the radiative pathway compared to the other molecules.^49,50^

### 2.2 Natural Transition Orbitals and Excited-State Symmetry Breaking

To investigate the nature of the electronic transitions observed in the 1PA spectra, we calculated the natural transition orbitals (NTOs) associated with the *S*_0_ *→ S*_1_ transition, as shown in Figure 3a. The optimized ground-state structures reveal that the samples have two axial branches, approximately aligned with the **DQ** core, and two oblique branches, tilted relative to the main axis and projected out of the central plane. These conformations are particularly evident in the lateral views shown in the insets of Figure 3a.

The NTOs indicate that the electronic transitions of the three compounds are of π-π* nature (see Figure 3a), consistent with our initial assumption. From a qualitative perspective, the hole orbital exhibits notable differences in electronic distribution among the compounds. In **DQ-IMD**, electronic delocalization mainly concentrates on the axial region, with negligible contribution from the oblique branches. **DQ-DDA** displays the opposite behavior: its electronic distribution localizes in the **DQ** core and two oblique branches. In contrast, **DQ-DPA** exhibits broader electronic delocalization, covering almost the entire molecular structure. On the other hand, the particle orbital displays very similar characteristics in all three samples, revealing an electronic redistribution, with a reduction in the ED branches and an increase in the **DQ** moiety, which extends residually over the phenyl rings.

The dihedral angles (see Table 2) between the branches (axial and oblique) and the **DQ** core provide insights into the differences in electronic distribution observed in the samples (the definitions of the dihedral angles δ_1_, δ_2/3_, and δ_4/5_ are illustrated in Figure 3b. The ground-state structures of the samples are nonplanar due to steric hindrance between ED branches, resulting in dihedral angles δ_1_, δ_2/3_, and δ_4/5_ to deviate significantly from 180°. However, we highlight a relevant trend: ED branches with dihedral angles less deviated from 180° are those that contribute to the electronic delocalization in the hole orbital (hereafter referred to as electronically active branches). This effect occurs because more planar conformations favor better electronic overlap, enhancing the interaction between the ED units and the **DQ** core.^5,50,51^ In this context, **DQ-DPA** exhibits a slightly higher degree of structural alignment, likely due to the rotational flexibility of the **DPA** moiety, which allows the molecule to adopt a more planar conformation. This behavior supports the redshift observed in the 1PA spectrum compared to other molecules (see Figure 1a-c), in agreement with the initial assumption.

**Table 2:** Structural and Electronic Parameters of the Diquinoxaline Derivatives, Including the Dihedral Angles (δ_1_, δ_2/3_, and δ_4/5_), the Transferred Charge (*q*_CT_), the Effective Charge Displacement Length (*D*_CT_), the Lippert–Mataga Slope $\left(\Delta \mathrm{\nu ̅}/\Delta F_{LM}\right)$ ), the Difference between the Permanent Dipole Moment of the First Excited State and the Ground One $\left(|\Delta {\mathrm{\mu ⃗}}_{01}|\right)$ , the Electronic Coupling between the Branches (*V*), the Sum of the Solvation and Coulomb Repulsion Energy (λ + γ), and the Ivanov Dipolar Parameter (*D*)

Samples	δ_1_ (°)	δ_2/3_ (°)	δ_4/5_ (°)	*q*_CT_ (|e^–^|)^a^	*D*_CT_ (*Å*)^a^	$\frac{\Delta \mathrm{\nu ̅}}{\Delta F_{LM}}\;\left(cm^{-1}\right)$	$\left|\Delta {\mathrm{\mu ⃗}}_{01}\right|\;\left(D\right)$		*V* (cm^–1^)	(λ + γ) (cm^–1^)	*D*
**DQ-IMD**	146	–146	–139	0.72	0.38	7000 ± 1000	1.30^a^	18 ± 3	527	3661	0.96
**DQ-DPA**	146	–145	–144	0.73	0.15	8000 ± 2000	0.54^a^	17 ± 3	632	4821	0.97
**DQ-DDA**	146	–139	–144	0.76	0.96	2600 ± 500	3.53^a^	10 ± 2	256	1570	0.94

a Properties determined by QCCs at the IEFPCM-TD-CAM-B3LYP/6-311++G­(d,p) level in the unrelaxed state.

The visual inspection of the NTOs suggests two general behaviors. First, the electronic excitation involves ICT from the ED branches (**IMD**, **DPA**, and **DDA**) to the EW **DQ** moiety. Second, both orbitals exhibit a symmetric charge distribution around the molecular structure, as expected for a quasi-centrosymmetric quadrupolar configuration. To gain a deeper understanding of these aspects, we determined the difference between the permanent dipole moment of the first excited state (*S*_1_) and the ground one (*S*_0_) ( $\left|\Delta {\mathrm{\mu ⃗}}_{01}\right|=\left|{\mathrm{\mu ⃗}}_{11}-{\mathrm{\mu ⃗}}_{00}\right|$ ) using two complementary approaches: Le Bahers’ metric,^52^ to determine $\left|\Delta {\mathrm{\mu ⃗}}_{01}\right|$ of the unrelaxed excited state (ground state geometry), and the solvatochromic analysis via the Lippert-Mataga equation,^53,54^ to estimate $\left|\Delta {\mathrm{\mu ⃗}}_{01}\right|$ of the relaxed excited state.

Based on Le Bahers’ metric, the compounds exhibit a high transferred charge (*q*_*CT*_), with an average value of 0.74 |*e* ^*‑*^|, confirming the pronounced ICT character of the **DQ** derivatives (see Table 2). The effective charge displacement length (*D*_*CT*_) is particularly low for **DQ-DPA** and **DQ-IMD**, ranging from 0.15 to 0.38 Å, respectively. This result shows that the centroids of positive and negative charge (*R*_+_ and *R*_–_; see Table SI5) are approximately superimposed and centered on the molecular structure. Consequently, the *D*_*CT*_ and *q*_*CT*_ values for **DQ-DPA** and **DQ-IMD** lead to a small $\left|\Delta {\mathrm{\mu ⃗}}_{01}\right|$ of approximately 0.92 D ( $\left|\Delta {\mathrm{\mu ⃗}}_{01}\right|=D_{CT}\cdot q_{CT}$ ),^52^ revealing a symmetrical charge distribution in the ground-state geometry, consistent with the quasi-centrosymmetric quadrupolar configuration in these molecules.

**DQ-DDA** displays a 3.6-fold increase in *D*_*CT*_ compared to the other samples. This increase suggests fluctuations in molecular structure and in the surrounding solvent orientation, even in the unrelaxed state.^55,56^ Consequently, **DQ-DDA** shows a higher $\left|\Delta {\mathrm{\mu ⃗}}_{01}\right|$ (3.53 D), indicating a slight asymmetry in the charge distribution pattern relative to the other two compounds. The extent of this asymmetry will be further analyzed in the next section. Moreover, this behavior may be one of the factors contributing to the significant Δν̅ observed for **DQ-DDA**, suggesting that the molecule undergoes a more pronounced solvation process and, consequently, a greater stabilization of the excited state,^57^ as evidenced in Figure 2d.

Figure 4a presents the solvatochromism measurements for the **DQ-IMD**, while the results for the other molecules are shown in Figure SI2. We observed that **DQ-IMD** exhibits almost no solvatochromism in absorption but displays a strong one in fluorescence emission, with increasing solvent polarity inducing a bathochromic shift of approximately 2520 cm^–1^ from toluene (a less polar solvent) to methanol (a more polar one). Additionally, Δν̅ increases linearly with the rise in solvent orientation polarizability (Δ*F*_*LM*_, see Figure 4b), indicating (i) a negligible contribution from specific solute–solvent interactions and (ii) a relaxed excited state with a strong dipolar character, an initially surprising result given the structure of the compound. The **DQ-DPA** and **DQ-DDA** molecules exhibit a similar behavior.

**Figure 4:**
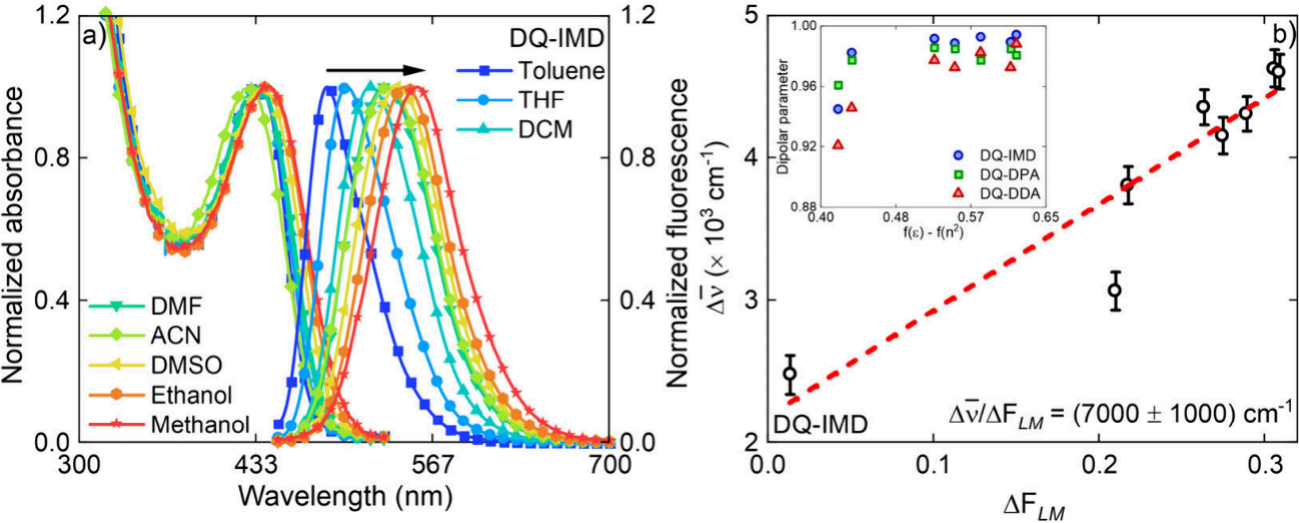
a) Normalized absorption and fluorescence spectra of **DQ-IMD** in different solvents; b) Stokes shift (Δν̅) as a function of solvent orientation polarizability (Δ*F*_LM_) for **DQ-IMD**. The inset in b) shows the dipolar parameter (*D*) as a function of solvent orientation polarizability for the diquinoxaline derivatives.

We determined the slope (Δν̅/ΔF_LM_, Table 2) by performing a linear regression of Δν̅ versus Δ*F*_*LM*_ for the **DQ** derivatives, which enabled the estimation of the experimental $\left|\Delta {\mathrm{\mu ⃗}}_{01}\right|$ (relaxed state) using the Lippert–Mataga equation. As shown in Table 2, the magnitude of $\left|\Delta {\mathrm{\mu ⃗}}_{01}\right|$ in the relaxed state increases approximately 23-fold, on average, for **DQ-IMD** and **DQ-DPA** compared to the unrelaxed state, while for **DQ-DDA**, this increase was smaller (∼2.8-fold). When comparing the samples, we observed that $\left|\Delta {\mathrm{\mu ⃗}}_{01}\right|$ follows the order: **DQ-IMD** > **DQ-DPA** > **DQ-DDA**. Therefore, these elevated $\left|\Delta {\mathrm{\mu ⃗}}_{01}\right|$ values suggest that the molecules undergo a symmetry-breaking process during excited-state relaxation, leading to a dipolar character.

The observations above can be summarized as follows: the ground state of all samples is essentially quadrupolar, as evidenced by the negligible solvatochromism observed in absorption.^55^ The same conclusion applies to the unrelaxed excited state of the molecules, except for minor conformational and electronic variations. However, during the excited-state relaxation, the compounds undergo symmetry-breaking, resulting in an asymmetric charge distribution, and becoming dipolar.

The symmetry-breaking effect in quadrupolar systems is a recurrent feature^41,55,56,58,59^ and has been thoroughly analyzed by Terenziani et al.^60^ In that study, the authors demonstrated that the excited state (*S*_1_) can be conditionally unstable. In sufficiently strong electronic–vibrational coupling conditions, the potential energy surface becomes bistable, with two equivalent minima corresponding to symmetry-broken structures with dipolar character. In the excited state, the system relaxes to one of these minima, which is then stabilized by polar solvents. Thus, symmetry breaking may arise from the cooperative action of electronic–vibrational coupling and polar solvation. Additionally, the symmetry-breaking effect and solvent influence can be more directly described by the model proposed by Ivanov,^61^ in which the extent of asymmetry is quantified by the dipolar parameter (*D*), defined as

1 $$D=\sqrt{1-\frac{4V^{2}}{{\left(\lambda +\gamma \right)}^{2}}}$$

in which *V* represents the electronic coupling between the branches (arising from dipole–dipole interaction), λ corresponds to the solvation energy, and γ refers to the Coulomb repulsion energy between the charges on the EW units. Section SI3 provides a more detailed discussion of eq 1 and the procedure used to determine the parameters *V*, λ, and γ, whose data are summarized in Table 2 for DCM.

In general, we found that *V* is significantly smaller than λ + γ for all samples. As a consequence, all of them exhibit a high dipolar parameter (*D* > 0.90), even in low-polarity solvents, with this value increasing with solvent polarity (see inset in Figure 4b). We also observed that **DQ-IMD** and **DQ-DPA** display higher *D* values than **DQ-DDA**, a trend consistent with their respective $\left|\Delta {\mathrm{\mu ⃗}}_{01}\right|$ . This is due to the smaller difference between the parameters *V* and λ + γ for **DQ-DDA** compared to the other molecules. Finally, we highlight that the low excitation anisotropy values stem from the symmetry-breaking process, which alters the angle between μ_01_ and μ_10_.

All the above observations indicate that the **DQ** derivatives exhibit an essentially quasi-centrosymmetric quadrupolar character and that symmetry breaking occurs after the vertical transition. We then analyze the 2PA of the compounds, with the additional aim of investigating what these data can reveal about the electronic symmetry in the unrelaxed state, given that, in quasi-centrosymmetric quadrupolar structures, 1PA and 2PA tend to be mutually exclusive.^55,62,63^

### 2.3 Multiphoton Absorption and Sum-Over-States Model

Figure 5a–d shows the 1PA, 2PA, and 3PA spectra of the **DQ** derivatives in DCM. We determined σ_2*PA*_ in the range of 550 to 1000 nm and the 3PA cross-section (σ_3*PA*_) from 1000 to 1180 nm using the absolute MPEF technique. The analysis of fluorescence intensity versus excitation power reveals slopes of approximately 2.1 ± 0.1 and 3.0 ± 0.1 on a logarithmic scale for two- and three-photon excitation (see Figure SI5a–f), respectively, confirming the pure multiphoton nature of the absorption.

**Figure 5:**
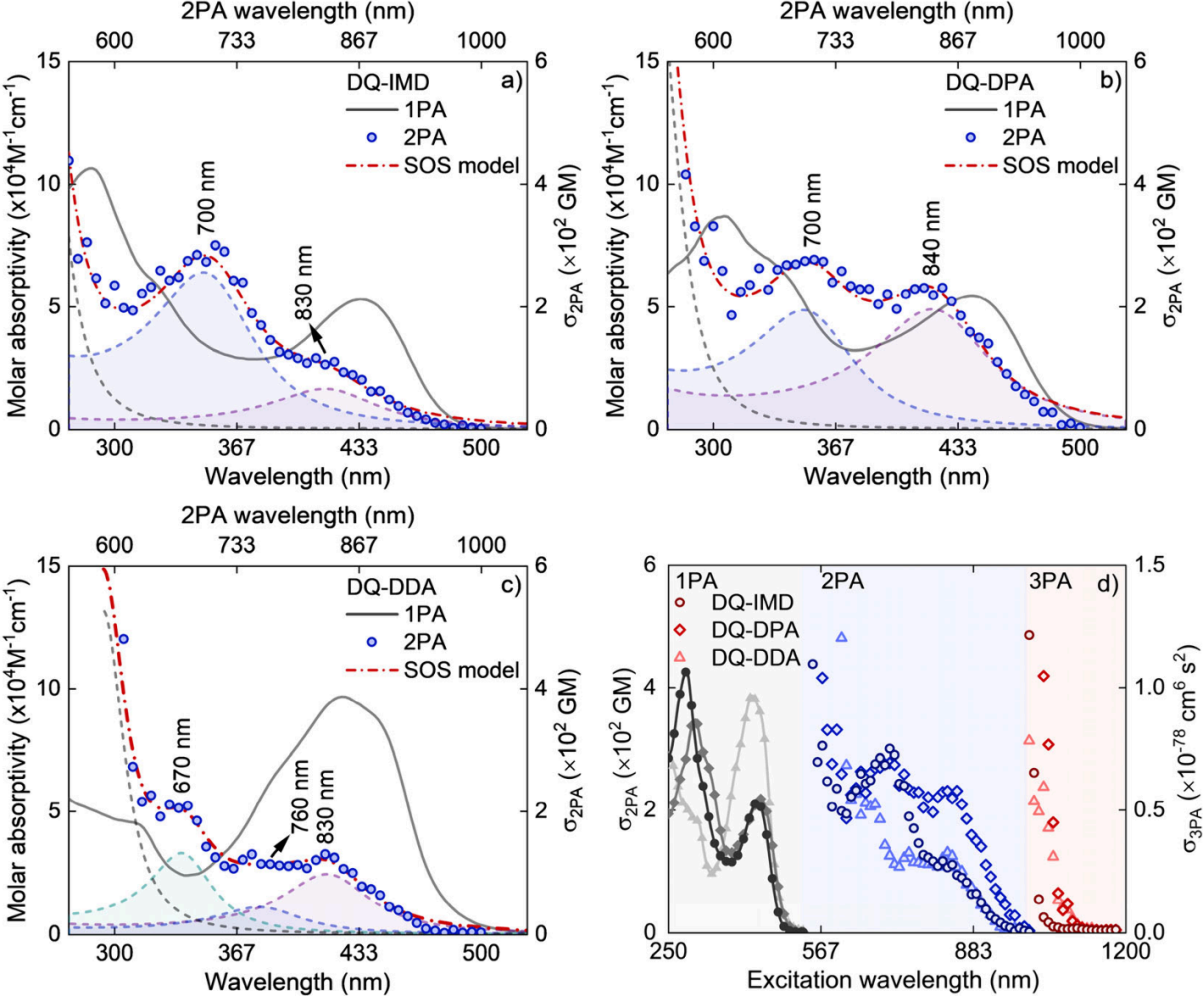
a–c) One-photon absorption (gray lines; left and bottom axes) and two-photon absorption (blue circles; right and top axes) spectra of diquinoxaline derivatives in DCM. The dashed curves represent bands obtained by the SOS model, while the red dash-dotted lines correspond to the sum of these curves. d) Comparative plot of one-, two-, and three-photon absorption (red symbols: right axis) spectra for all compounds. The shaded spectral regions correspond to one- (light gray), two- (light blue), and three-photon absorption (light red).

The 2PA spectra of the samples (see Figure 5a–c, blue circles) display well-defined 2PA bands, covering the visible to near-infrared spectral region. In addition, we observed a resonant enhancement effect at wavelengths shorter than 600 nm as the excitation wavelength approaches the lowest-energy 1PA band.^64^

By analyzing the **DQ-IMD** and **DQ-DPA** molecules, we identified two broad 2PA bands (shaded areas of light purple and light blue). The lower-energy band is located at *ca*. 835 nm with σ_2*PA*_ of around 170 GM, while the higher-energy band is centered at *ca*. 700 nm with σ_2*PA*_ of about 275 GM (see Table 3). We also observe that the maxima of the 1PA bands is shifted by approximately 0.4 eV relative to those of the 2PA bands; this most likely indicates that the latter are associated with distinct electronic states. Indeed, as previously discussed for **DQ-IMD** and **DQ-DPA**, $\left|\Delta {\mathrm{\mu ⃗}}_{01}\right|$ in the unrelaxed excited state is negligible due to the quasi-centrosymmetric quadrupolar configuration of these molecules. As a consequence, the one-photon allowed states become weakly two-photon allowed (and *vice versa*), following the dipole selection rule for 2PA. The results obtained through quadratic response function (QRF) calculations using the CAM-B3LYP/6-311++G­(d,p) approach (see Section SI5 and Table SI6) also support this inference, showing that excited states strongly two-photon allowed, *i.e*., with high 2PA transition probabilities, are indeed those that are weakly accessible via 1PA, as anticipated. Finally, we should highlight that this behavior is widely observed in quasi-centrosymmetric quadrupolar systems.^55,62,63,65,66^

**Table 3:** Two-Photon Absorption Properties of the **DQ** Derivatives in DCM, Including the Two-Photon Absorption Cross-Section (σ^2PA^), the Transition Dipole Moment from States *g* to *e* $\left(|{\mathrm{\mu ⃗}}_{ge}|\right)$ , and the Transition Dipole Moment from States *e* to *e*_*n*_^′^ ( $\left|{\mathrm{\mu ⃗}}_{ee_{n}^{\prime }}\right|$ for *n* = 1, 2, 3, and 4)

Samples	σ_(1)_^2PA^ (GM)^a^	σ_(2)_^2PA^ (GM)^a^	σ_(3)_^2PA^ (GM)^a^	$$\left|{\mathrm{\mu ⃗}}_{ge}\right|\left(D\right)$$	$|{\mathrm{\mu ⃗}}_{ee_{\left(1\right)}^{\prime }}|\left(D\right)$ ^b^	$|{\mathrm{\mu ⃗}}_{ee_{\left(2\right)}^{\prime }}|\left(D\right)$ ^b^	$|{\mathrm{\mu ⃗}}_{ee_{\left(3\right)}^{\prime }}|\left(D\right)$ ^b^	$|{\mathrm{\mu ⃗}}_{ee_{\left(4\right)}^{\prime }}|\left(D\right)$ ^b^
**DQ-IMD**	110 ± 20	280 ± 60		8.0	11	16	10	
**DQ-DPA**	230 ± 50	270 ± 50		8.0	18	14	10	
**DQ-DDA**	120 ± 20	110 ± 20	200 ± 40	12.0	8	5	6	8

a The subscript in σ_(*n*)_^2PA^ corresponds to the maximum 2PA cross-section of the first (σ_(1)_^2PA^), second (σ_(2)_^2PA^), and third (σ_(3)_^2PA^) 2PA band.

b The subscript in $\left|{\mathrm{\mu ⃗}}_{ee_{\left(n\right)}^{\prime }}\right|$ corresponds to the first (*e*_(1)_^′^), second (*e*_(2)_^′^), and third (*e*_(3)_^′^) 2PA bands. Observation $\left|{\mathrm{\mu ⃗}}_{ee_{\left(3\right)}^{\prime }}\right|$ describes the resonant enhancement of **DQ-IMD** and **DQ-DPA**, and $\left|{\mathrm{\mu ⃗}}_{ee_{\left(4\right)}^{\prime }}\right|$ describes the resonant enhancement of **DQ-DDA**.

For the **DQ-DDA** molecule, we identified three spectral regions with distinct behaviors, which we associated with three 2PA bands (shaded areas of light purple, light blue, and light green). The two lower-energy bands are centered at *ca*. 830 and 760 nm, exhibiting σ_2*PA*_ of approximately 115 GM (see Table 3). The higher-energy band at *ca*. 660 nm shows a greater σ_2*PA*_ of around 200 GM. Unlike the other two molecules, **DQ-DDA** exhibits a slight asymmetry in the charge distribution (even in the unrelaxed condition), which should, in principle, relax the 2PA selection rule. However, we observe some behaviors that suggest the opposite: (i) the maximum of the lower-energy 2PA band exhibits a blue shift of approximately 0.1 eV relative to its 1PA counterpart, and (ii) the band at 660 nm lies in the valley of the 1PA spectrum. This behavior indicates that the states that are strongly two-photon allowed differ from those one-photon allowed. The results of the QRF-CAM-B3LYP calculations also support this inference, showing that states with low 1PA oscillator strength characterize the three 2PA bands. Thus, despite the slight asymmetric charge distribution of **DQ-DDA**, the observed transitions do not fully follow the behavior expected for systems with strong dipolar character, indicating that the quasi-centrosymmetric quadrupolar nature prevails, as also observed in other studies.^62,67–69^

To gain a better understanding of the 2PA process in **DQ** derivatives, we employed the *sum-over-states* (SOS) model, treating the system as a quasi-three-level system.^70^ This approach is particularly suitable for centrosymmetric molecules (or quasi-centrosymmetric).^5,70–74^ The model includes a ground state (*g*), a one-photon allowed intermediate state (*e*), and a two-photon allowed final state (*e* ^′^). To describe systems with multiple 2PA accessible states, additional states *e*_*n*_^′^ are considered (hence the term ‘quasi-three-level’).^70^ This model is described by

2 $$\sigma_{2PA}=\frac{2}{5}\frac{{\left(2\pi \right)}^{4}}{{\left(chn\right)}^{2}}L^{4}\frac{1}{\pi }\underset{e^{\prime }}{\sum }\frac{\nu }{{\left(\nu_{ge}-\nu \right)}^{2}+\Gamma_{ge}^{2}}\cdot \frac{\left({\left|{\mathrm{\mu ⃗}}_{ee^{\prime }}\right|}^{2}\cdot {\left|{\mathrm{\mu ⃗}}_{ge}\right|}^{2}\right)\cdot \Gamma_{ge^{\prime }}}{{\left(\nu_{ge^{\prime }}-2\nu \right)}^{2}+\Gamma_{ge^{\prime }}^{2}}$$

In Eq. 2, *c*, *h*, *n*, and *L* correspond to the speed of light, Planck’s constant, the refractive index (1.4242 for DCM), and the Onsager local field factor, respectively. The terms ν, ν_*ge*_, $\nu_{ge^{\prime }}$ , Γ_*ge*_, and $\Gamma_{ge^{\prime }}$ represent, respectively, the laser excitation frequency, the transition frequencies from states *g*→*e* and *g*→*e* ^′^, and the FWHM of each transition. $\left|{\mathrm{\mu ⃗}}_{ee^{\prime }}\right|\cdot \left|{\mathrm{\mu ⃗}}_{ge}\right|$ is the three-state term, where $\left|{\mathrm{\mu ⃗}}_{ee^{\prime }}\right|$ is the transition dipole moment from states *e* to *e* ^′^, involved in the 2PA process. It is worth mentioning that $\left|{\mathrm{\mu ⃗}}_{ge}\right|$ , Γ_*ge*_, and ν_*ge*_ were determined from the 1PA spectra (see Section SI2), while the remaining parameters were obtained by fitting Eq. 2. The data are summarized in Table 3 ( $\Gamma_{ge^{\prime }}∼0.26eV$ ).

Figure 5a–c shows the 2PA spectra fitted using the SOS model (red dash-dotted lines). We employed a two-excited states system for **DQ-IMD** and **DQ-DPA**, while for **DQ-DDA**, we used a model with three excited states; in all cases, we included an additional term to account for resonant enhancement. Overall, the quasi-three-level model adequately describes the experimental data and indicates strong coupling between the *e* and *e*_(*n*)_^′^ states. This good agreement further supports previous observations that the states active in 2PA differ from those accessed via 1PA. Furthermore, it is worth noting that although some 2PA spectral regions overlap with 1PA bands, Terenziani et al.^60^ demonstrated that this behavior is due to the vibronic activation of one-photon-allowed states, a phenomenon commonly observed in quasi-centrosymmetric quadrupolar molecules.^60,75–82^ In the case of **DQs**, this effect becomes even more pronounced due to the weak electronic coupling among the branches (*V*), resulting in a slight separation between the states accessible via 1- and 2PA.^61,83^

The comparison of 2PA results shows that **DQ-DPA** exhibits the best overall performance. In the lower-energy band, where the resonant enhancement effect does not significantly influence, **DQ-DPA** displays σ_2*PA*_ of 230 GM, while **DQ-IMD** and **DQ-DDA** show similar values of about 115 GM (within experimental error; see Table 3). The analysis of NTOs for this 2PA band (see Figure SI6) indicates that this behavior arises from the number of ED branches involved in the electronic delocalization: four in **DQ-DPA** and only two in **DQ-IMD** and **DQ-DDA**, as also shown in Figure 3a. When normalizing σ_2*PA*_ by the number of active ED branches in each molecule, we obtain a value of approximately 58 ± 6 GM. These results suggest that the three ED branches (**IMD**, **DPA**, and **DDA**) contribute similarly to the 2PA response and indicate a possible additive effect with respect to the number of electronically active branches. The additive effect is consistent with the weak electronic coupling between the branches, which leads them to contribute almost independently to the global 2PA.^27,29^

In conclusion, the primary influence of ED units on 2PA is related to the molecular conformation. The ED branches that allow the molecule to adopt a more planar geometry (limited by steric hindrance) lead to a greater number of electronically active branches, thus extending the electron delocalization and, consequently, increasing σ_2*PA*_; this is likely the case for the **DPA** moiety, as evidenced by the NTO analysis and dihedral angle discussed.

Finally, Figure 5d shows the 3PA spectra of the compounds, which reveal two spectral regions. At wavelengths shorter than 1050 nm, we observe a progressive increase in σ_3*PA*_, attributed to the resonant enhancement effect by 2PA. At wavelengths longer than 1050 nm, however, the σ_3*PA*_ values are significantly lower (∼0.02 × 10^−78^ cm^6^ s^2^ photons^–2^ at 1100 nm) and remain relatively constant up to 1180 nm. This spectral region is weakly accessible via 1PA and, consequently, by 3PA since both processes involve mutually allowed states, which explains the behavior of 3PA in this range.^84^

### 2.4 Multiphoton Polymerization

Given the good multiphoton absorption performance of the **DQ** derivatives, we evaluated their efficiency as two-photon PIs. To this end, we prepared a mixture of two acrylic resins in equal weight proportions (see Section SI6), incorporating the **DQ** compounds in different concentrations (∼0.05–0.22 wt %). Among the various methods available to evaluate PI efficiency, in this study we chose to determine the 2PP threshold fluence by fabricating 3D microstructures. We define this threshold as the minimum energy required to trigger 2PP.

Figure 6a shows the threshold fluence for different concentrations of the **DQ** derivatives, ranging from 0.05 to 0.22 wt %. We observed a progressive decrease in threshold fluence values for the three lowest concentrations (0.05, 0.09, and 0.13 wt %), from approximately 0.0030 J/cm^2^ (0.09 nJ) to 0.0015 J/cm^2^ (0.05 nJ). At the two highest concentrations (0.18 and 0.22 wt %), the behavior suggests a possible saturation, with values remaining nearly constant around 0.0013 J/cm^2^ (0.04 nJ). Among the three samples, there is a slight tendency for higher efficiency in **DQ-DPA**, possibly related to its larger σ^2*PA*^ at *ca*. 775 nm.

**Figure 6:**
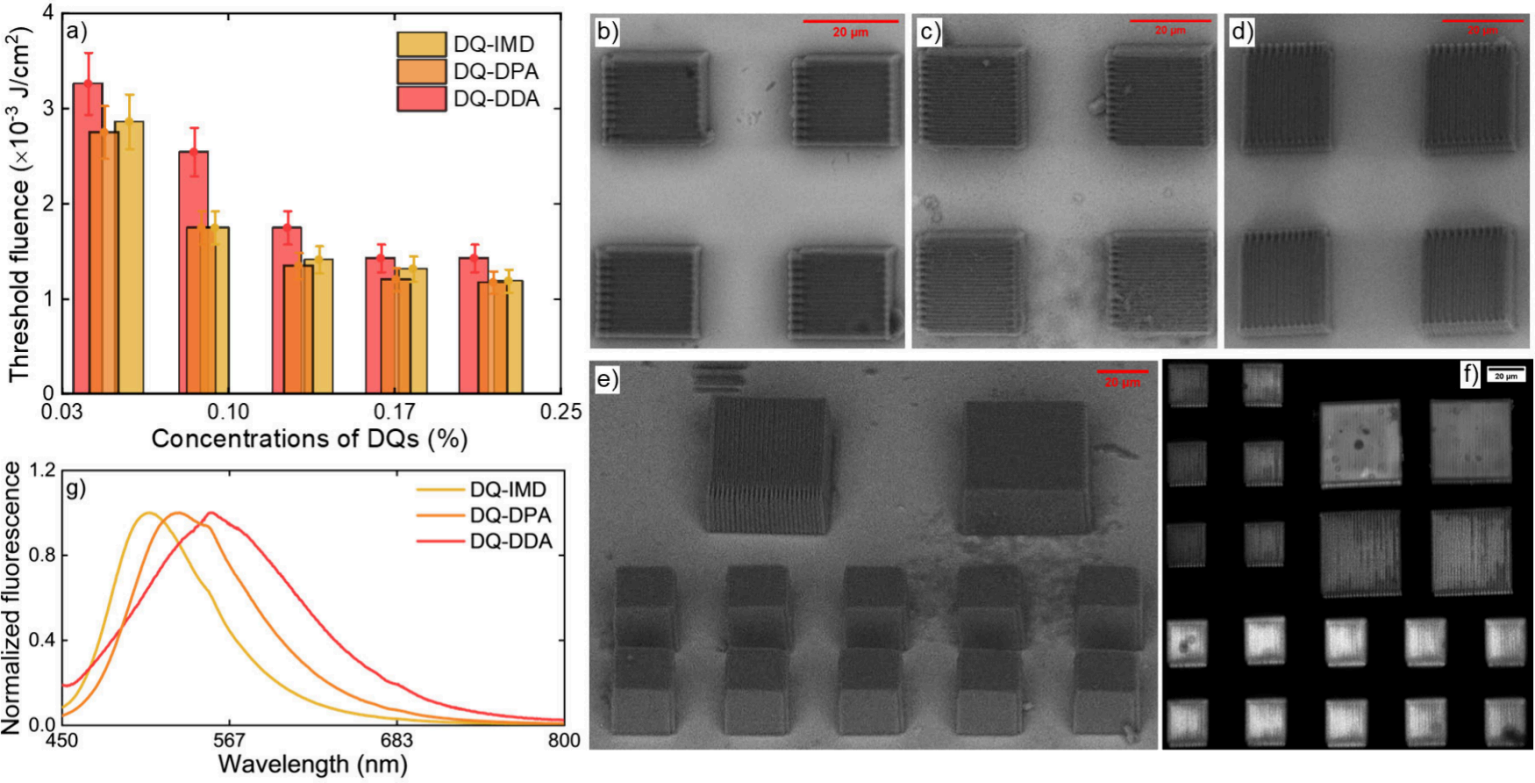
a) Threshold fluence versus the concentration of diquinoxalines mixed with two acrylic resins in an equal mass ratio. Scanning electron microscopy images of the microstructures fabricated by two-photon polymerization using b) **DQ-IMD**, c) **DQ-DPA**, and d, e) **DQ-DDA** as the photoinitiator (scale bar: 20 μm). f) Confocal fluorescence image of microstructures fabricated by two-photon polymerization using **DQ-DDA** as the photoinitiator (scale bar: 20 μm). g) Fluorescence emission spectra of microstructures fabricated by two-photon polymerization using the diquinoxalines as photoinitiators.

When we compare the 2PP results with benchmark PIs such as Irgacure 369,^20,21^ Irgacure TPO-L^22,23^ and BDEBP,^85^ which exhibit σ_2*PA*_ values of up to 6 GM at 800 nm, we observe that the **DQs** show comparable or superior performance, with higher cross sections and lower polymerization threshold energies, even operating at concentrations up to ∼ 4 times lower (see Table 4). In addition, several studies have reported PIs with diverse molecular architectures exhibiting σ_2*PA*_ values in the range of 52 to 401 GM (at *ca*. 800 nm, see Table 4).^7,20,85,86^ In these compounds, the 2PP threshold energies range from 0.08 to 5.0 nJ, and concentrations range from 0.08 to 1.0 wt %. Although some of these compounds exhibit larger σ_2*PA*_ values than the **DQ** derivatives, our results indicate that the latter act as efficient PIs, achieving low polymerization thresholds at significantly reduced concentrations.

**Table 4:** Two-Photon Polymerization Properties of **DQ** Derivatives and Other Photoinitiators. The Reported Parameters Include the Solvent, The Two-Photon Absorption Cross-Section (σ_2PA_) at 800 nm, the Polymerization Threshold Energy, And the Photoinitiator Concentration

Sample	Solvent^a^	σ_2PA_ (GM)^a^	Threshold energy (nJ)	PI concentration (wt %)
**DQ-IMD**	DCM	120 ± 20	0.04	0.23
**DQ-DPA**	DCM	200 ± 30	0.04	0.22
**DQ-DDA**	DCM	110 ± 20	0.05	0.21
Irgacure TPO-L^b^ ^,^ ^c^	Ethanol^b^	<1^b^	0.06^c^	0.60^c^
Irgacure 369^d^ ^,^ ^e^	Methanol^d^	<5^d^	35.00^e^	0.24^e^
BDEBP^f^	1-Propanol^f^	6^f^	0.13^f^	1.00^f^
DR13^g^	Ethanol^g^	52^g^	0.50^g^	1.00^g^
HBO NBu_2_ ^h^	DCM^h^	80^h^	0.08^h^	0.08^h^
B3K^e^	Tetrahydrofuran^e^	238^e^	5.00^e^	0.31^e^
R2^e^	Tetrahydrofuran^e^	314^e^	5.00^e^	0.34^e^
R1^e^	Tetrahydrofuran^e^	318^e^	5.00^e^	0.38^e^
6B^f^	DCM^f^	401^f^	0.13^f^	1.00^f^

a Solvent used to dissolve the photoinitiators for the measurement of the two-photon absorption cross-section (σ_2PA_) determined at 800 nm.

b Previously reported by Mendonca et al.^22^

c Previously reported by Baldacchini et al.^23^

d Previously reported by Schafer et al.^21^

e Previously reported by Pucher et al.^20^

f Previously reported by Nazir et al.^85^

g Previously reported by Otuka et al.^86^

h Previously reported by Valverde et al.^7^

Finally, Figure 6b-f presents microscopy images of the microstructures fabricated via 2PP, using ∼ 0.22 wt % of the **DQ** derivatives and an intensity of ∼ 13 GW/cm^2^. We observe that the structures exhibit good structural integrity, as indicated by qualitative analysis and intrinsic fluorescence emission (see Figure 6f-g), the latter arising from residual, nonactivated molecules that retain their photophysical properties within the structures. Consequently, these results demonstrate the viability of **DQs** as two-photon PIs; furthermore, the observed intrinsic fluorescence enables their application in photonic devices such as high-resolution displays, 3D data storage, anticounterfeiting systems, and devices with tunable emission.^24–26^

## 3 Conclusion

In summary, we determined the linear photophysical properties and the 2- and 3PA processes of three **DQ** derivatives functionalized with different electron-donating branches (**IMD**, **DPA**, and **DDA**), resulting in molecular structures with quasi-centrosymmetric quadrupolar character. Finally, considering their high absorption cross sections, we evaluated the performance of these compounds as PIs for 2PP.

The results demonstrated that the studied compounds exhibit broad 1PA in the 250–510 nm range, associated with multiple electronic transitions, as evidenced by excitation anisotropy spectra and the QCC results. Regarding emissive properties, we observed fluorescence emission between 520 and 558 nm, with ϕ_fl_ ranging from 3% to 42% and τ_fl_ between 2 and 8 ns for the samples. Analysis of NTOs, combined with Le Bahers’ metric, indicated significant ICT and negligible $\left|\Delta {\mathrm{\mu ⃗}}_{01}\right|$ in the unrelaxed excited state, reflecting the quasi-centrosymmetric quadrupolar configuration of the compounds. However, during excited-state relaxation, symmetry breaking occurs, making the molecules dipolar, with a *D* close to 0.97. Based on Ivanov’s model, we demonstrated that this effect stems, in part, from weak electronic coupling between electron-donating branches (*V*).

Regarding 2PA, the **DQ** derivatives exhibited σ_2*PA*_ of ∼150 GM for the lower-energy bands and ∼250 GM for the higher-energy bands. The 2PA bands exhibit spectral shifts relative to the 1PA ones, a behavior attributed to the dipole selection rule for (quasi-)­centrosymmetric molecules, in which strongly one-photon allowed states are weakly two-photon allowed and *vice versa*. This interpretation was supported by QCC results and by good agreement with the SOS model based on a quasi-three-level system. We also observed that the three electron-donating branches (**IMD**, **DPA**, and **DDA**) exhibit comparable 2PA responses, with additive behavior as the number of electronically active branches increases. In this context, **DQ-DPA** exhibited the best 2PA performance, as the **DPA** branch promotes more planar molecular conformations (limited by steric hindrance), enabling greater electronic delocalization throughout the molecule. Additionally, we observed σ_3*PA*_ of ∼0.02 × 10^−78^ cm^6^ s^2^ photons^–2^ at 1100 nm.

Finally, we confirmed the viability of the **DQ** derivatives as two-photon PIs by fabricating 3D microstructures with inherent emissive properties. Overall, the three compounds performed comparably, with **DQ-DPA** showing slightly superior behavior. These results help clarify the structure–property relationships in **DQ**-based quadrupolar systems, with potential applications as a multiphoton PI.

## 4 Experimental Details

In the Supporting Information (SI) section, we describe all the details about the measurements performed, the equipment used, and the procedures for the quantum chemical calculations.

## Supplementary Material



## Data Availability

The authors confirm that the data supporting the conclusions of this study are available in the article and its SI.

## Notes

Synthesis details (Section SI1); experimental procedures for linear photophysical characterization, including one-photon absorption, fluorescence emission, fluorescence quantum yield, excitation anisotropy, solvatochromism, and fluorescence lifetime (Section SI2); Ivanov model and symmetry-breaking analysis (Section SI3); multiphoton-excited fluorescence methodology for two- and three-photon absorption (Section SI4); quantum-chemical calculation procedures and results, including equilibrium geometries, transition properties, and Le-Bars’ parameters (Section SI5); two-photon polymerization methodology (Section SI6); NMR spectra of all compounds (Section SI7); and supplementary references (Section SI8) (PDF)
