# Supplementary material for: Multiphoton Absorption in Branched Quadrupolar Diquinoxalines for Two-Photon Polymerization Design
Source: ACS Phys Chem Au. 2026 May 29;6(4):803–14. doi: 10.1021/acsphyschemau.6c00035 (PMC13397456; doi:10.1021/acsphyschemau.6c00035)
Supplement: Supplementary file 1 [file pg6c00035_si_001.pdf]

Multiphoton Absorption in Branched Quadrupolar Diquinoxalines for Two-Photon Polymerization Design

João V. P. Valverde[a,*], Welisson de P. Silva[b], Daniel L. Silva[c], Nícolas O. Decarli[b], Mieczysław Łapkowski[b], Leonardo De Boni[a], and Cleber R. Mendonça[a,*]

[a] São Carlos Institute of Physics, University of São Paulo, CP 369, 13560-970 São Carlos, SP, Brazil
[b] Faculty of Chemistry, Silesian University of Technology, CP 44-100, Gliwice, GLW, Poland.
[c] Department of Natural Sciences, Mathematics and Education, Federal University of São Carlos, Rod. Anhanguera – Km 174, 13600-970 Araras, SP, Brazil

*Author to whom correspondence should be addressed: joaovalverde@ifsc.usp.br and crmendon@ifsc.usp.br

Supplementary Information

SI1 Synthesis details

The synthetic route to obtain the final compounds are shown in Scheme 1. DQ-Br was synthesized by condensation reaction of 4,4′-dibromobenzil under reflux in acetic acid being isolated as gray solid. The next step was based on a nitrogen-carbon coupling reaction catalyzed by palladium (Buchwald-Hartwig cross-coupling reaction) using different donor units (DPA, DDA and IMD) to obtain the final compounds DQ-DPA, DQ-DDA, DQ-IMD in good yields after purification by chromatography column and precipitation in methanol.

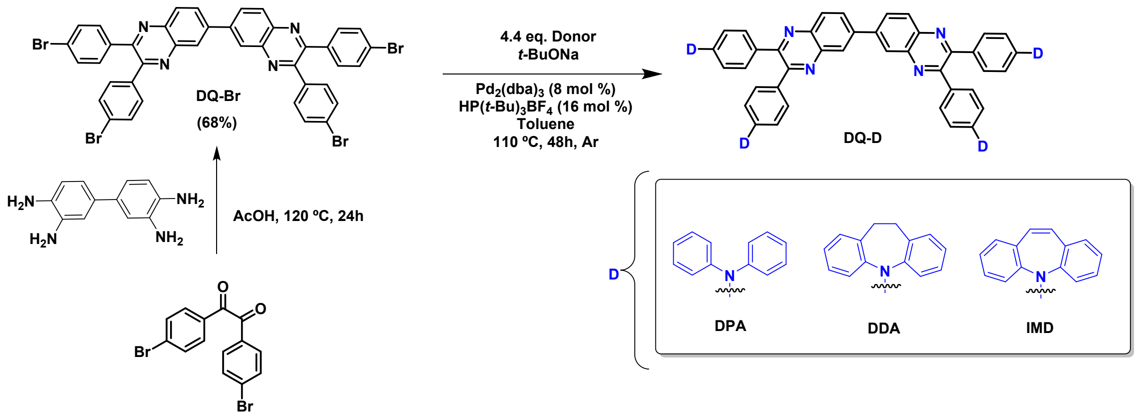

Scheme 1. Synthetic route to obtain the **DQ**-Donors.

2,2',3,3'-Tetrakis(4-bromophenyl)-6,6'-biquinoxaline (**DQ-Br**)

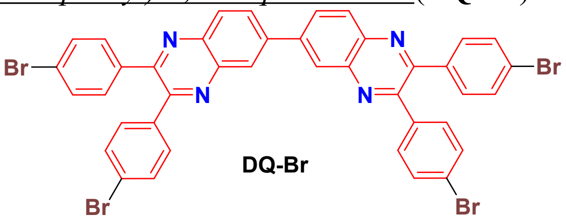

3,3'-diaminobenzidine (0.30 g, 1.3 mmol) and 4,4'-dibromobenzil (1.00 g, 2.72 mmol) were uniformly mixed in 30 mL of glacial acetic acid under argon atmosphere. The mixture was heated at 120 °C for 24h with stirring. Then, the mixed solution was poured into distilled water and the gray precipitate was collected by filtration, washed several times with water, and dried in a vacuum. The solid was dissolved in 200 mL of chloroform and washed two times with sodium hydroxide solution (1 M), brine and distilled water. The organic layer was dried with $MgSO_4$ and evaporated. The solid obtained was crystallized in acetone/methanol to obtain **DQ-Br** in 68% yield as a gray solid. ^{1}H NMR (300 MHz, $CDCl_3$) δ/ppm: 8.57 (d, J = 1.9 Hz, 12H), 8.31 (d, J = 8.8 Hz, 2H), 8.25 (dd, J = 8.8, 2.0 Hz, 2H), 7.54 (d, J = 8.6 Hz, 4H), 7.45 (d, J = 8.4 Hz, 4H).

General procedure for N-C coupling in the DQ-Br core

The mixture of **DQ-Br** (0.227 mmol), the respective electron donor (1.138 mmol), *t*-BuONa (1.57 mmol), $Pd_2(dba)_3$ (0.027 mmol), HP(*t*-Bu)$_3$BF$_4$ (0.054 mmol) and 25 mL of toluene was stirred at 110 °C for 48h under argon atmosphere. The progress of the reaction was monitored by TLC to ensure the total consumption of the core. The hot mixture was filtered through celite and washed with dichloromethane and ethyl acetate. The residue was purified by column chromatography by gradient of pure chloroform and chloroform /ethyl acetate (3:1) as eluent. Compounds were crystallized in methanol to give the pure materials.

4,4',4'',4'''-([6,6'-biquinoxaline]-2,2',3,3'-tetrayl)tetrakis(N,N-diphenylaniline) (**DQ-DPA**)

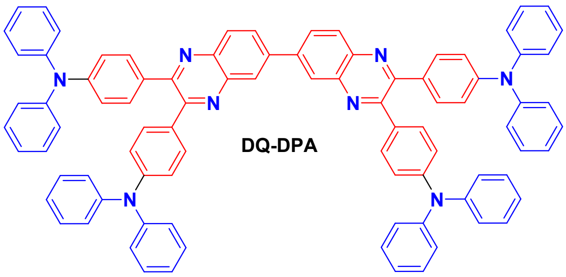

Yellow solid (55% of yield). ^{1}H NMR (300 MHz, $CDCl_3$) δ/ppm: 8.53 (d, *J* = 1.9 Hz, 2H), 8.25 (d, *J* = 8.7 Hz, 2H), 8.17 (dd, *J* = 9.1, 1.5 Hz, 2H), 7.49 (d, *J* = 8.8 Hz, 8H), 7.34 – 7.26 (m, 16H), 7.18 – 7.12 (m, 16H), 7.11 – 7.02 (m, 16H). ^{13}C NMR (75 MHz, $CDCl_3$) δ/ppm: 153.81, 153.37, 148.67, 147.30, 141.32, 140.91, 140.80, 132.42, 130.83, 129.70, 129.39, 129.09, 127.19, 125.05, 123.53, 122.11. HRMS: m/z calcd for $C_{88}H_{63}N_8$ $(M+H)^+$: 1231.5176; found: 1231.5609.

5,5',5'',5'''-([6,6'-biquinoxaline]-2,2',3,3'-tetrayltetrakis(benzene-4,1-diyl))tetrakis(5H-dibenzo[b,f]azepine) **(DQ-IMD)**

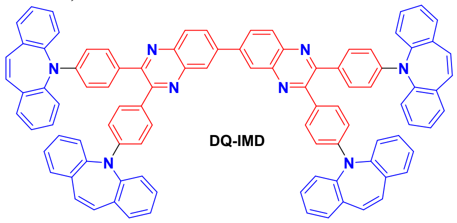

Orange solid (52% of yield). ^{1}H NMR (300 MHz, $CDCl_3$) δ 8.26 (dd, *J* = 7.9, 3.1 Hz, 3H), 8.13 (d, *J* = 7.0 Hz, 1H), 8.08 (d, *J* = 8.2 Hz, 1H), 7.97 (*d*, J = 8.3 Hz, 1H), 7.67 (d, *J* = 7.9 Hz, 4H), 7.63 – 7.38 (m, 36H), 7.19 (dd, *J* = 4.3, 3.1 Hz, 4H), 6.95 (d, *J* = 3.5 Hz, 4H), 6.84 (s, 4H), 6.68 – 6.58 (m, 4H). ^{13}C NMR (75 MHz, $CDCl_3$) δ 154.49, 152.54, 152.30, 148.30, 147.78, 145.06, 144.53, 143.60, 143.42, 141.75, 141.64, 140.72, 139.99, 138.96, 138.84, 136.08, 135.98, 135.92, 135.86, 130.76, 130.74, 130.61, 130.57, 130.39, 130.30, 130.22, 129.70, 129.62, 129.07, 128.98, 128.04, 127.90, 127.70, 127.65, 127.46, 127.41, 125.73, 125.45, 123.89, 123.67, 121.83, 121.70, 121.19, 120.65, 120.44, 115.08, 114.27, 111.77, 111.34. HRMS: m/z calcd for $C_{95}H_{75}N_8$ $(M+H)^+$: 1327.6115; found: 1327.5861.

5,5',5'',5'''-([6,6'-biquinoxaline]-2,2',3,3'-tetrayltetrakis(benzene-4,1-diyl))tetrakis (10,11-dihydro-5H-dibenzo[b,f]azepine) **(DQ-DDA)**

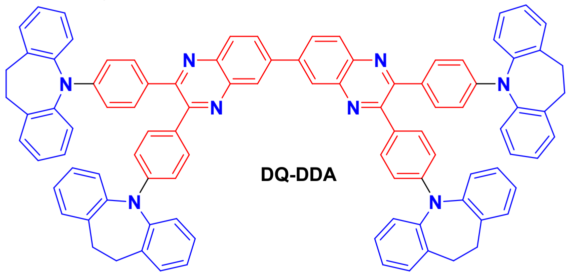

Yellow solid (44% of yield). ^{1}H NMR (300 MHz, CD_2Cl_2) δ 8.62 (dd, *J* = 9.5, 3.1 Hz, 2H), 8.32 (dd, *J* = 6.0, 1.6 Hz, 1H), 8.26 – 8.16 (m, 2H), 8.08 (d, *J* = 8.1 Hz, 1H), 7.63 – 7.47 (m, 11H), 7.46 – 7.30 (m, 34H), 7.23 (dd, *J* = 17.5, 8.1 Hz, 3H), 3.22 (s, 8H), 3.12 (s, 8H). ^{13}C NMR (75 MHz, $CDCl_3$) δ 152.70, 148.31, 147.70, 145.57, 145.46, 145.33, 144.81, 143.76, 142.20, 142.14, 140.59, 139.28, 137.96, 137.91, 137.27, 136.40, 131.49, 131.37, 131.15, 131.11, 129.25, 129.18, 128.41, 128.38, 128.07, 128.02, 127.81, 127.69, 127.59, 127.54, 127.48, 125.94, 125.72, 124.17, 121.87, 121.40, 121.18, 114.71, 113.98, 30.84, 30.71.

SI2 Linear photophysical properties

To determine the linear photophysical properties of diquinoxaline (**DQ)** derivatives, we prepared solutions of the sample in dichloromethane (DCM) at approximately 10^{-5} mol/L. We used a spectrometer (Shimadzu, UV-Vis 1800) and a fluorimeter (Hitachi, F-7000) to record one-photon absorption (1PA) and steady-state fluorescence spectra, respectively. We placed the solutions in quartz cells with optical path lengths of 2.0 mm (for absorption) and 10.0 mm (for fluorescence). The fluorescence spectra for different excitation wavelengths are shown in **Fig. SI1**. Using the well-known Brouwer method[1,2], we obtained the fluorescence quantum yield (ϕ_{fl}), employing Coumarin 500 dissolved in methanol (ϕ_{fl} = 46%)[3] as the standard sample. We measured the excitation anisotropy spectrum using the L-configuration[4], with two polarizers positioned along the detection and excitation channels. To minimize rapid molecular rotation, we prepared a DCM solution saturated with polystyrene[5]. Additionally, we performed solvatochromism measurements by dissolving the samples in various solvents, including toluene, tetrahydrofuran (THF), dimethylformamide (DMF), ethanol, acetonitrile (ACN), and methanol (see absorption and fluorescence spectra in **Fig. SI2**). We use the refractive index (n) and dielectric constant (ε) from the

literature to calculate the solvent orientation polarizability (ΔF_{LM})[6,7]. Finally, we carried out time-resolved fluorescence measurement to determine the fluorescence lifetime (τ_{fl}) of the **DQs**. For this purpose, we used a homemade setup described in detail in Ref.[8,9] The time-resolved fluorescence decay curves are shown in **Fig. SI3**.

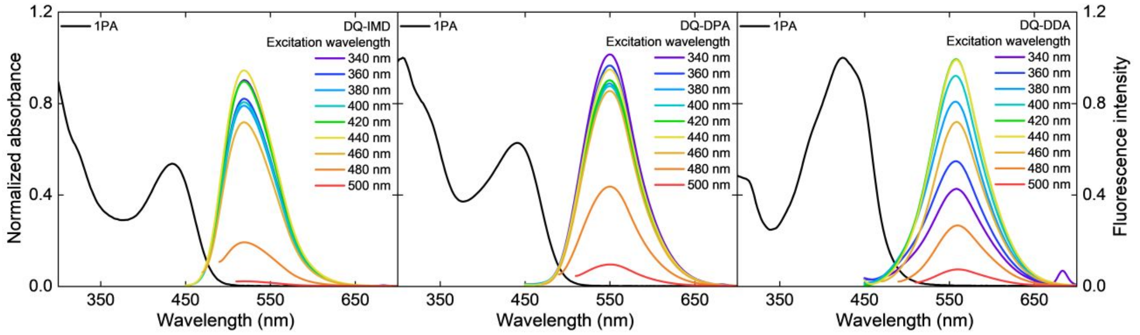

Figure SI1 – Normalized one-photon absorption and fluorescence emission spectra for different excitation wavelengths of **DQ** derivatives.

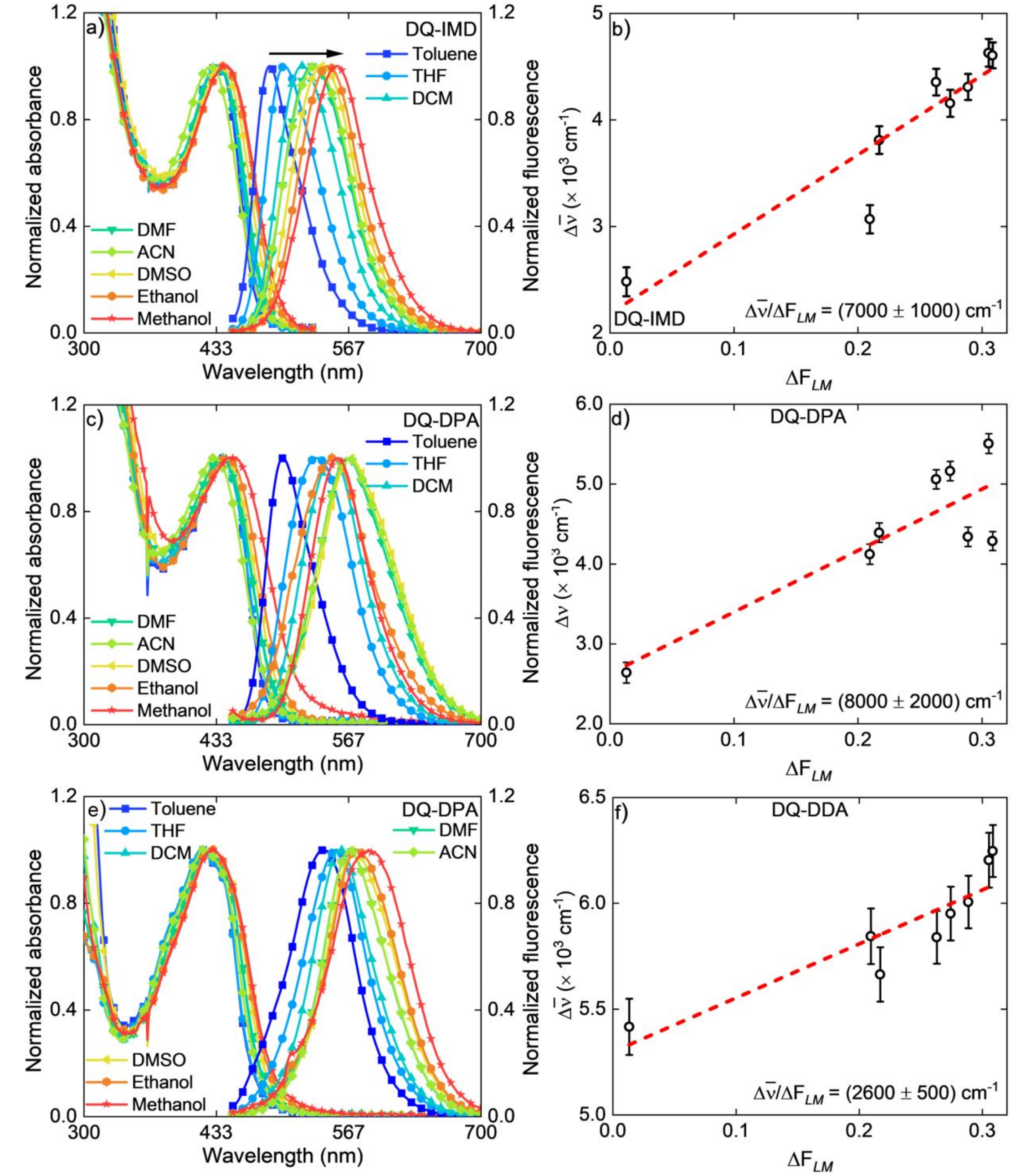

Figure SI2 – a, c, and e) Normalized absorption and fluorescence spectra of **DQs** in different solvents; b, d, and f) Stokes shift ($\Delta\bar{\nu}$) as a function of solvent orientation polarizability (ΔF_{LM}) for **DQs**. The red dashed line corresponds to the linear fit used to estimate the slope ($\Delta\bar{\nu}/\Delta F_{LM}$).

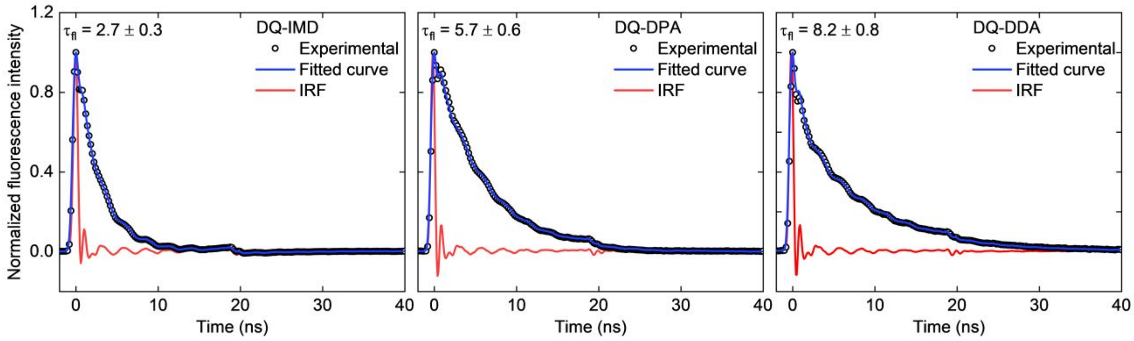

Figure SI3 – Fluorescence intensity as a function of time (black circles), convolution-fitted curve (blue line), and instrument response function (IRF: red line) of the **DQ** derivatives.

We decomposed the 1PA spectrum into Gaussian functions to estimate the transition dipole moment from the ground state to the first excited one ($|\vec{\mu}_{01}|$), as shown in **Fig. SI4**. The gray and blue curves correspond to the individual Gaussians, while the dashed pink curve represents their overlap. From the decomposed spectrum and using the equation below[10], we estimated the value of $|\vec{\mu}_{01}|$:

$$|\vec{\mu}_{01}|^2 = 3\ln(10)\left(\frac{10}{2\pi}\right)^3 \frac{hcn}{N_A L^2} \int \frac{\varepsilon(\omega)}{\omega} d\omega\,. \tag{1}$$

In this equation, h is Planck's constant, c is the light speed, n is the refractive index of the medium ($n = 1.4241$ for DCM), N_A is Avogadro's constant, and $L = \frac{3n^2}{2n^2+1}$ is the Onsager local field factor[10]. For the estimation of $|\vec{\mu}_{01}|$, we used only the blue hatched curve in **Fig. SI4a-c**, which is expected to correspond to the S_0–S_1 transition, as also supported by the theoretical results (considering the correction of the applied energy redshift of ~0.45 eV, see **Section SI4**). The results are summarized in **Table SI4**.

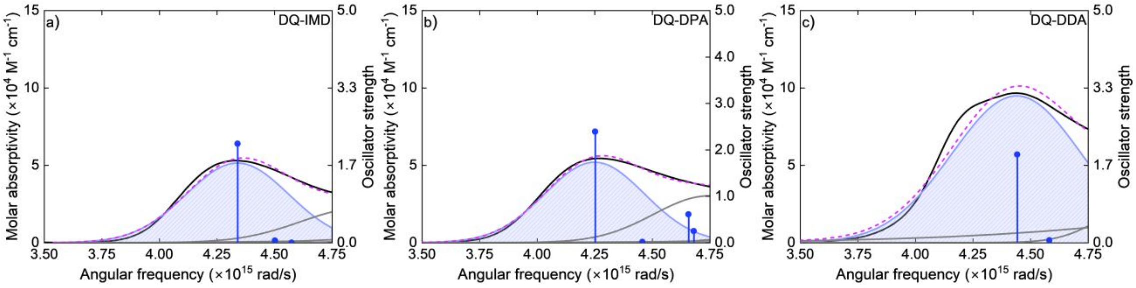

Figure SI4 – One-photon absorption spectra (black lines: left axis and bottom axis) of the **DQs** decomposed into Gaussian curves (gray and blue lines). The dashed pink line represents the overlap of the Gaussian curves. The blue vertical sticks with dots on top (right axis and bottom axis) correspond to the oscillator strengths obtained from IEFPCM:TD-CAM-B3LYP/6-311++G(d,p) calculations, which were uniformly redshifted in energy by 0.47, 0.42, and 0.46 eV for **DQ-IMD**, **DQ-DPA**, and **DQ-DDA**, respectively.

Table SI1 – Transition dipole moment from the ground state to the first excited state ($|\vec{\mu}_{01}|$), $S_0 \rightarrow S_1$ transition frequency (ω_{01}), $S_0 \rightarrow S_1$ linewidth (FWHM: Γ_{01}), and corresponding integral ($\int \frac{\varepsilon}{\omega} d\omega$).

Sample	$|\vec{\mu}_{01}|$ (D)	ω_{01} $(\times 10^{15})$	Γ_{01} $(\times 10^{14}\ rad/s)$	Integral $(M^{-1}cm^{-1})$
**DQ-IMD**	7.8	4.34	5.26	6733
**DQ-DPA**	7.8	4.25	5.11	6713
**DQ-DDA**	11.7	4.36	6.62	15155

SI3 Symmetry breaking properties

As discussed in the main text, Ivanov *et al.*[11] proposed a model to quantify the effect of symmetry breaking in quadrupolar molecules. This model considers only the electronic coupling between the branches V, the solvation energy λ, and the Coulomb repulsion energy γ between the charges localized on the left and right sides of the electron-withdrawing moieties ($\delta_{L,R} = e|a_{L,R}|^2$), with the condition $|a_L|^2 + |a_R|^2 = 1$. We can then characterize the degree of asymmetry of a molecule by the dipolar parameter D, defined as[11]:

$$D = |a_L^2 - a_R^2|, \tag{2}$$

or, in its most usual form

$$D = \sqrt{1 - \frac{4V^2}{(\lambda + \gamma)^2}}. \tag{3}$$

The dipolar parameter ranges from 0, when there is no symmetry breaking, to 1, in the case of complete symmetry breaking (*i.e.*, the case of a purely dipolar molecule in the excited state). To calculate it, we need to determine the terms in Eq. (3). In practice, we can estimate V from the energy difference between the lowest energy band in 1PA ($S_0 \rightarrow S_1$) and 2PA ($S_0 \rightarrow S_2$) spectra, which is equivalent to the Davydov splitting[11–13]. On the other hand, $\lambda + \gamma$ can be expressed as[11]:

$$\lambda + \gamma = 2(\Delta E_{\text{fl}} + V). \tag{4}$$

where ΔE_{fl} represents the shift of the fluorescence band from a nonpolar solvent ($D = 0$) to a polar solvent ($D \neq 0$)[11,12] . In our analysis, we calculate ΔE_{fl} relative to toluene. Furthermore, we assume that V remains approximately constant across all solvents, as it can only be estimated for DCM, where both 1PA and 2PA spectra are available (See **Table 2** for values). The remaining data are summarized in **Table SI2**.

Table SI2 – Fluorescence emission wavenumber ($\bar{\nu}_{fl}$), fluorescence band shift (ΔE_{fl}) relative to toluene, sum of the solvation energy and the Coulomb repulsion energy ($\lambda + \gamma$), and the dipolar parameter (D) of the **DQ** derivatives.

**DQ-IMD**				
Solvent	$\bar{\nu}_{\mathrm{fl}}\ (cm^{-1})$	$\Delta E_{\mathrm{fl}}\ (cm^{-1})$	$(\lambda + \gamma)\ (cm^{-1})$	D
Toluene	20534	---	---	---
THF	19920	614	2282	0.887
DCM	19231	1303	3661	0.958
DMSO	18501	2032	5120	0.979
DMF	18832	1701	4458	0.972
Ethanol	18315	2219	5493	0.981
ACN	18762	1772	4599	0.973
Methanol	18018	2516	6087	0.985
**DQ-DPA**				
Solvent	$\bar{\nu}_{\mathrm{fl}}\ (cm^{-1})$	$\Delta E_{\mathrm{fl}}\ (cm^{-1})$	$(\lambda + \gamma)\ (cm^{-1})$	D
Toluene	19960	---	---	---
THF	18709	1251	3767	0.942
DCM	18182	1778	4821	0.965
DMSO	17590	2370	6004	0.978
DMF	17668	2292	5849	0.976
Ethanol	18182	1778	4821	0.965
ACN	17668	2292	5849	0.976
Methanol	17986	1974	5213	0.970
**DQ-DDA**				
Solvent	$\bar{\nu}_{\mathrm{fl}}\ (cm^{-1})$	$\Delta E_{\mathrm{fl}}\ (cm^{-1})$	$(\lambda + \gamma)\ (cm^{-1})$	D
Toluene	18450	---	---	---
THF	18051	400	1311	0.921
DCM	17921	529	1570	0.945
DMSO	17498	952	2416	0.977
DMF	17606	845	2201	0.973
Ethanol	17331	1119	2750	0.983
ACN	17606	845	2201	0.973
Methanol	17036	1414	3340	0.988

SI4 Two- and three-photon absorption measurements

We performed two- and three-photon absorption (2PA and 3PA) measurements using the multiphoton-excited fluorescence technique[14–16], whose detailed description of the experimental setup can be found in Ref.[17]. In this technique, the fluorescence intensity excited by multiphoton absorption is calibrated relative to that obtained from 1PA in the same sample and under identical experimental conditions[14–17]. Briefly, we

employed a regeneratively amplified Yb:KGW femtosecond laser system (Pharos PH1, Light Conversion, *Inc.*) with the following specifications: 800 μJ pulse energy, 240 fs pulse duration at 1030 nm, and a repetition rate of 7.5 kHz. We utilized this laser system to pump an optical parametric amplifier (OPA; Orpheus, Light Conversion, *Inc.*), which is capable of generating tunable pulses in the 220–3000 nm range, with pulse durations ranging from 100-180 fs. We measured 2PA in the range of 550-1000 nm and 3PA between 1000-1180 nm, both with a spectral step of 10 nm. We prepared sample solutions in DCM at concentrations similar to those used for 1PA measurements (~ 10^{-5} mol/L) and place them in quartz cells with an optical path length of 10 mm.

Figures SI5a–c presents the fluorescence emission spectra obtained under one- (400 nm), two- (700 and 830 nm), and three-photon (1100 nm) excitation, which show good agreement among them. **Fig. SI5d–f** displays the fluorescence intensity as a function of the excitation power on a logarithmic scale. Linear regression of the data reveals that, for 2PA (700 and 830 nm, represented by blue circles and yellow triangles, respectively), the slope is 2.1 ± 0.1. In contrast, for 3PA (1100 nm, represented by red diamonds), the slope is 3.0 ± 0.1, confirming the multiphoton nature of the absorption process.

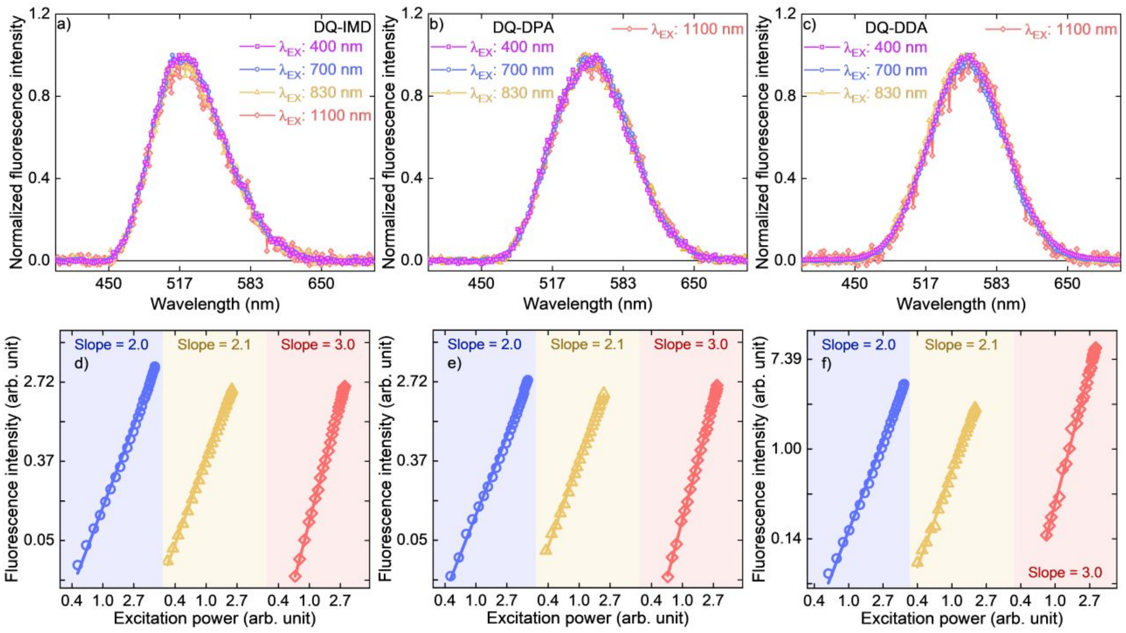

Figure SI5 – a–c) Fluorescence emission spectra of **DQ** derivatives in DCM, obtained under one- (400 nm), two- (700 and 830 nm), and three-photon (1100 nm) excitation. d–f) Fluorescence intensity dependence on excitation power on a logarithmic scale. Blue circles and yellow triangles correspond to the curves obtained from 2PA (excitation wavelength: 700 and 830 nm), while red diamonds represent 3PA (excitation wavelength: 1100 nm). Blue, yellow, and red lines correspond to linear regressions, with slopes indicated in the graphs.

SI5 Quantum chemical calculation procedures

We performed quantum chemical calculations (QCCs) within the framework of density functional theory (DFT) and its time-dependent extension (TD-DFT) using the Gaussian 09 package[18]. We can divide the procedure employed in this work into two steps: 1) geometry optimization and vibrational frequency calculations, in which we used the B3LYP[19] hybrid exchange-correlation functional with the Pople's 6-311G(d,p) basis set[20]. No imaginary vibrational modes (negative frequency) were found, which means that optimized structures corresponding to a stable energy minimum were obtained; 2) TD-DFT calculations, in which we used the CAM-B3LYP[21] hybrid exchange-correlation functional combined with the 6-311G++(d,p) basis set to determine the 20 lowest-energy singlet electronic transitions by 1PA, the natural transition orbitals (NTOs)[22] of the desired electronic transitions, and the Le Bahers' metrics[23], such as: transferred charge (q_{CT}), effective charge displacement length (D_{CT}) and difference in permanent dipole moment between the first excited state and the ground one ($D_{CT} \cdot q_{CT} = |\Delta\vec{\mu}_{01}| = |\vec{\mu}_{11}| - |\vec{\mu}_{00}|$). We carried out all calculations taken into account the solution environment (in this case DCM solvent) by using the polarizable continuum model with integral equation formalism variant (IEF-PCM)[24,25].

To calculate the 2PA transition probability of the 10 lowest-energy singlet electronic transitions, we employed the quadratic response function (QRF)[26] within the DFT framework, as implemented in the DALTON package[27]. For this purpose, we employ the CAM-B3LYP functional with the 6-311++G(d,p) basis set. Due to computational limitations, we performed these calculations only in gas phase.

We summarize all the obtained results, including optimized geometry, electronic transitions by 1PA, Le Bahers' parameters, and electronic transitions by 2PA, in **Tables SI3**, **SI4**, **SI5**, and **SI6**, respectively.

Table SI3 – Optimized structures of **DQs** obtained through IEFPCM-B3LYP/6-311G(d,p) calculations in DCM medium.

**DQ-IMD**				**DQ-DPA**				**DA-DDA**			
IEFPCM-TD-B3LYP/6-311G(d,p)											
Atom	x (Å)	y (Å)	z (Å)	Atom	x (Å)	y (Å)	z (Å)	Atom	x (Å)	y (Å)	z (Å)
C	2.2125	-2.1430	1.2286	C	2.2466	-2.1070	1.3804	C	2.2467	-2.1079	1.3343
C	3.3171	-1.3298	0.8753	C	3.3362	-1.2758	1.0241	C	3.3342	-1.2759	0.9763
C	3.0810	0.0184	0.4932	C	3.0803	0.0687	0.6428	C	3.0805	0.0716	0.6038
C	1.7658	0.5333	0.5015	C	1.7563	0.5621	0.6548	C	1.7557	0.5640	0.6211
C	0.6924	-0.2656	0.8578	C	0.6967	-0.2546	1.0119	C	0.6971	-0.2546	0.9759
C	0.9425	-1.6234	1.2181	C	0.9680	-1.6083	1.3719	C	0.9680	-1.6095	1.3316
N	4.5806	-1.8152	0.9475	N	4.6083	-1.7392	1.0935	N	4.6072	-1.7435	1.0328
C	5.5898	-1.0317	0.6055	C	5.6028	-0.9402	0.7490	C	5.5945	-0.9405	0.6885
C	5.3304	0.2955	0.0735	C	5.3276	0.3839	0.2147	C	5.3269	0.3920	0.1741
N	4.1120	0.7970	0.0690	N	4.0969	0.8622	0.2158	N	4.0951	0.8708	0.1832

C	6.9466	-1.5812	0.8285	C	6.9648	-1.4796	0.9878	C	6.9640	-1.4833	0.9112
C	6.3763	1.1603	-0.5424	C	6.3527	1.2679	-0.3958	C	6.3650	1.2763	-0.4034
C	7.3030	0.6523	-1.4607	C	7.3525	0.7844	-1.2521	C	7.3888	0.8030	-1.2362
C	8.2243	1.4918	-2.0789	C	8.2329	1.6480	-1.8887	C	8.3028	1.6629	-1.8261
C	8.2499	2.8604	-1.7873	C	8.1606	3.0340	-1.6738	C	8.2430	3.0537	-1.5988
C	7.3203	3.3693	-0.8746	C	7.1659	3.5223	-0.8104	C	7.2186	3.5315	-0.7536
C	6.3897	2.5335	-0.2682	C	6.2729	2.6548	-0.2008	C	6.2999	2.6614	-0.1900
C	7.1857	-2.9504	0.6374	C	7.2112	-2.8458	0.7838	C	7.2449	-2.8125	0.5721
C	8.4249	-3.5171	0.8868	C	8.4475	-3.4066	1.0631	C	8.5011	-3.3575	0.8145
C	9.4929	-2.7324	1.3686	C	9.4852	-2.6224	1.5944	C	9.5019	-2.5936	1.4186
C	9.2483	-1.3627	1.5935	C	9.2389	-1.2579	1.8185	C	9.2167	-1.2744	1.7862
C	8.0076	-0.8070	1.3205	C	8.0066	-0.6991	1.5102	C	7.9657	-0.7241	1.5295
C	10.7087	-4.8125	4.0904	C	9.9614	-4.9522	3.4286	C	12.2642	-5.4619	0.5920
C	11.1743	-3.6044	4.4716	C	10.8206	-4.5237	2.4077	C	12.8639	-5.5046	2.0036
C	11.9324	-2.6501	3.6647	N	10.7387	-3.1938	1.9027	C	12.0560	-4.8064	3.0843
C	11.7779	-2.5422	2.2680	C	11.9400	-2.4552	1.6999	C	11.0988	-3.7796	2.8827
N	10.7511	-3.2919	1.6077	C	12.9334	-2.4369	2.6877	N	10.8009	-3.1874	1.6219
C	10.9437	-4.7117	1.5836	C	12.1517	-1.7528	0.5063	C	11.7332	-3.0040	0.5486
C	10.8548	-5.4478	2.7822	C	13.3327	-1.0423	0.3114	C	12.4206	-4.0953	-0.0080
C	11.1793	-5.3575	0.3692	C	14.3234	-1.0316	1.2932	C	11.9171	-1.7283	-0.0047
C	11.2898	-6.7435	0.3186	C	14.1174	-1.7352	2.4795	C	12.7688	-1.5324	-1.0871
C	11.1637	-7.4910	1.4914	C	10.0464	-6.2527	3.9170	C	13.4643	-2.6098	-1.6305
C	10.9481	-6.8498	2.7037	C	10.9973	-7.1386	3.4107	C	13.2888	-3.8766	-1.0817
C	12.8845	-1.8227	4.2890	C	11.8602	-6.7115	2.4020	C	12.2646	-5.2728	4.3906
C	13.6795	-0.9556	3.5520	C	11.7704	-5.4177	1.8962	C	11.5612	-4.8020	5.4904
C	13.5412	-0.8905	2.1639	C	11.1581	2.9579	-1.4812	C	10.5912	-3.8264	5.2820
C	12.5913	-1.6832	1.5277	C	10.4175	3.5403	-2.5181	C	10.3714	-3.3294	4.0057
C	10.2769	6.4555	-1.8344	N	9.0584	3.9154	-2.3153	C	11.4977	3.8848	-0.6387
C	9.4299	6.6686	-2.8575	C	8.6184	5.1963	-2.7572	C	11.3078	5.4115	-0.5705
C	8.9918	5.7211	-3.8817	C	9.3991	6.3348	-2.5170	C	10.0965	6.0325	-1.2556
C	8.9093	4.3264	-3.6665	C	7.4061	5.3343	-3.4466	C	9.1163	5.3390	-1.9872
N	9.1904	3.7714	-2.3812	C	6.9817	6.5885	-3.8752	N	9.1733	3.9169	-2.1791
C	10.4711	3.9324	-1.7715	C	7.7640	7.7193	-3.6409	C	10.3610	3.4095	-2.8099
C	10.9755	5.2204	-1.4808	C	8.9755	7.5832	-2.9640	C	11.5405	3.3712	-2.0547
C	11.2128	2.8084	-1.3889	C	12.4885	2.6022	-1.6840	C	10.3459	2.9963	-4.1409
C	12.4214	2.9363	-0.7097	C	13.1040	2.8327	-2.9143	C	11.5162	2.5233	-4.7320
C	12.9225	4.2019	-0.4132	C	12.3706	3.4182	-3.9459	C	12.6966	2.4776	-3.9920
C	12.1997	5.3239	-0.7979	C	11.0359	3.7640	-3.7555	C	12.7078	2.9050	-2.6644
C	8.6180	6.2307	-5.1372	C	-1.7563	-0.5621	0.6548	C	9.9654	7.4274	-1.1588
C	8.1832	5.4037	-6.1650	C	-3.0803	-0.0687	0.6428	C	8.9094	8.1156	-1.7407
C	8.1133	4.0297	-5.9475	C	-3.3362	1.2758	1.0241	C	7.9454	7.4139	-2.4653
C	8.4812	3.5011	-4.7130	C	-2.2466	2.1070	1.3804	C	8.0613	6.0371	-2.5923
C	-1.7658	-0.5332	0.5016	C	-0.9680	1.6083	1.3720	C	-1.7555	-0.5641	0.6211
C	-3.0810	-0.0183	0.4932	C	-0.6967	0.2546	1.0119	C	-3.0803	-0.0717	0.6039
C	-3.3172	1.3298	0.8752	N	-4.0970	-0.8622	0.2158	C	-3.3340	1.2758	0.9766
C	-2.2126	2.1431	1.2285	C	-5.3276	-0.3840	0.2148	C	-2.2465	2.1077	1.3347
C	-0.9426	1.6235	1.2180	C	-5.6028	0.9401	0.7489	C	-0.9678	1.6093	1.3319
C	-0.6925	0.2657	0.8578	N	-4.6083	1.7392	1.0935	C	-0.6969	0.2545	0.9760
N	-4.1121	-0.7970	0.0691	C	-6.3527	-1.2680	-0.3957	N	-4.0949	-0.8708	0.1832
C	-5.3304	-0.2955	0.0736	C	-6.9648	1.4796	0.9877	C	-5.3267	-0.3920	0.1741
C	-5.5899	1.0318	0.6055	C	-7.2113	2.8458	0.7836	C	-5.5943	0.9404	0.6888
N	-4.5806	1.8153	0.9474	C	-8.4476	3.4066	1.0628	N	-4.6070	1.7434	1.0333
C	-6.3764	-1.1603	-0.5422	C	-9.4852	2.6225	1.5943	C	-6.3649	-1.2762	-0.4036
C	-6.9466	1.5812	0.8285	C	-9.2389	1.2580	1.8187	C	-6.9639	1.4831	0.9115
C	-7.1858	2.9504	0.6371	C	-8.0066	0.6992	1.5103	C	-7.2448	2.8123	0.5727
C	-8.4250	3.5172	0.8865	C	-7.3525	-0.7845	-1.2521	C	-8.5010	3.3572	0.8151
C	-9.4929	2.7326	1.3684	C	-8.2329	-1.6481	-1.8886	C	-9.5018	2.5931	1.4191
C	-9.2483	1.3629	1.5935	C	-8.1606	-3.0341	-1.6737	C	-9.2166	1.2740	1.7865
C	-8.0076	0.8071	1.3206	C	-7.1659	-3.5224	-0.8103	C	-7.9655	0.7238	1.5298
C	-7.3031	-0.6524	-1.4606	C	-6.2730	-2.6549	-0.2007	C	-7.3886	-0.8026	-1.2363
C	-8.2243	-1.4920	-2.0787	C	-11.1581	-2.9584	-1.4806	C	-8.3027	-1.6624	-1.8264
C	-8.2499	-2.8606	-1.7871	C	-10.4176	-3.5403	-2.5178	C	-8.2430	-3.0532	-1.5993
C	-7.3203	-3.3694	-0.8743	N	-9.0585	-3.9155	-2.3151	C	-7.2186	-3.5312	-0.7542
C	-6.3897	-2.5336	-0.2680	C	-8.6185	-5.1964	-2.7572	C	-6.2999	-2.6613	-0.1904
C	-10.2775	-6.4554	-1.8339	C	-9.3993	-6.3348	-2.5174	C	-11.4978	-3.8839	-0.6392
C	-9.4301	-6.6689	-2.8567	C	-7.4060	-5.3343	-3.4463	C	-11.3081	-5.4106	-0.5711
C	-8.9915	-5.7217	-3.8810	C	-6.9817	-6.5885	-3.8751	C	-10.0969	-6.0318	-1.2563

C	-8.9089	-4.3270	-3.6660	C	-7.7641	-7.7192	-3.6412	C	-9.1166	-5.3384	-1.9878
N	-9.1903	-3.7717	-2.3809	C	-8.9758	-7.5832	-2.9646	N	-9.1734	-3.9162	-2.1796
C	-10.4712	-3.9323	-1.7717	C	-12.4885	-2.6025	-1.6832	C	-10.3611	-3.4086	-2.8104
C	-10.9759	-5.2202	-1.4808	C	-13.1041	-2.8327	-2.9135	C	-11.5405	-3.3701	-2.0552
C	-11.2129	-2.8081	-1.3895	C	-12.3708	-3.4178	-3.9453	C	-10.3458	-2.9953	-4.1413
C	-12.4218	-2.9357	-0.7107	C	-11.0360	-3.7637	-3.7551	C	-11.5161	-2.5220	-4.7324
C	-12.9232	-4.2011	-0.4141	C	-9.9611	4.9521	3.4285	C	-12.6965	-2.4762	-3.9924
C	-12.2004	-5.3233	-0.7983	C	-10.8205	4.5237	2.4078	C	-12.7078	-2.9037	-2.6649
C	-8.6174	-6.2317	-5.1362	N	-10.7387	3.1939	1.9026	C	-9.9660	-7.4267	-1.1596
C	-8.1821	-5.4050	-6.1640	C	-11.9399	2.4553	1.6995	C	-8.9101	-8.1150	-1.7415
C	-8.1120	-4.0309	-5.9468	C	-12.9335	2.4368	2.6872	C	-7.9461	-7.4134	-2.4661
C	-8.4803	-3.5021	-4.7126	C	-12.1514	1.7531	0.5058	C	-8.0618	-6.0366	-2.5931
C	-10.7087	4.8127	4.0901	C	-13.3324	1.0426	0.3106	C	-12.2653	5.4616	0.5938
C	-11.1743	3.6046	4.4714	C	-14.3233	1.0317	1.2923	C	-12.8656	5.5024	2.0052
C	-11.9324	2.6504	3.6645	C	-14.1174	1.7351	2.4787	C	-12.0571	4.8043	3.0855
C	-11.7780	2.5424	2.2678	C	-10.0460	6.2525	3.9171	C	-11.0992	3.7783	2.8834
N	-10.7511	3.2921	1.6075	C	-10.9972	7.1385	3.4113	N	-10.8009	3.1868	1.6223
C	-10.9437	4.7119	1.5833	C	-11.8603	6.7114	2.4028	C	-11.7326	3.0040	0.5484
C	-10.8548	5.4480	2.7819	C	-11.7706	5.4177	1.8968	C	-12.4205	4.0955	-0.0075
C	-11.1793	5.3577	0.3689	H	2.4530	-3.1284	1.6772	C	-11.9156	1.7287	-0.0061
C	-11.2898	6.7437	0.3184	H	1.6017	1.5866	0.3393	C	-12.7667	1.5334	-1.0891
C	-11.1636	7.4912	1.4911	H	0.1467	-2.2473	1.6726	C	-13.4625	2.6109	-1.6319
C	-10.9480	6.8500	2.7034	H	7.4258	-0.2767	-1.4548	C	-13.2880	3.8772	-1.0819
C	-12.8845	1.8230	4.2888	H	8.9761	1.2498	-2.5679	C	-12.2658	5.2700	4.3921
C	-13.6796	0.9559	3.5518	H	7.0955	4.5860	-0.6213	C	-11.5620	4.7992	5.4916
C	-13.5413	0.8907	2.1637	H	5.5029	3.0492	0.4503	C	-10.5912	3.8245	5.2826
C	-12.5914	1.6834	1.5275	H	6.4181	-3.4680	0.3886	C	-10.3712	3.3282	4.0062
H	2.4014	-3.1677	1.5257	H	8.6154	-4.4589	0.8717	H	2.4545	-3.1306	1.6257
H	1.6290	1.5602	0.1854	H	10.0150	-0.6375	2.2486	H	1.6003	1.5900	0.3108
H	0.1108	-2.2493	1.5176	H	7.8466	0.3522	1.7137	H	0.1474	-2.2493	1.6322
H	7.3016	-0.4028	-1.7042	H	9.2305	-4.2642	3.8358	H	7.4685	-0.2555	-1.4509
H	8.9274	1.0764	-2.7901	H	12.7740	-2.9748	3.6144	H	9.0678	1.2542	-2.4717
H	7.3352	4.4288	-0.6486	H	11.3922	-1.7694	-0.2659	H	7.1471	4.5880	-0.5360
H	5.6645	2.9407	0.4253	H	13.4840	-0.5089	-0.6202	H	5.5153	3.0572	0.4433
H	6.3771	-3.5784	0.2841	H	15.2449	-0.4837	1.1349	H	6.4714	-3.4152	0.1128
H	8.5670	-4.5748	0.7130	H	14.8766	-1.7306	3.2535	H	8.7147	-4.3820	0.5339
H	10.0310	-0.7321	1.9918	H	9.3748	-6.5690	4.7072	H	9.9738	-0.6789	2.2821
H	7.8629	0.2474	1.5205	H	11.0655	-8.1484	3.7982	H	7.7669	0.2990	1.8228
H	10.2654	-5.4371	4.8606	H	12.6004	-7.3915	1.9956	H	11.2070	-5.7454	0.6336
H	11.0719	-3.3438	5.5211	H	12.4355	-5.0949	1.1044	H	12.7715	-6.1974	-0.0366
H	11.2510	-4.7622	-0.5334	H	10.6901	2.7894	-0.5189	H	13.8762	-5.0848	1.9602
H	11.4617	-7.2385	-0.6299	H	10.3355	6.2368	-1.9814	H	12.9840	-6.5477	2.3105
H	11.2376	-8.5718	1.4581	H	6.8010	4.4578	-3.6444	H	11.3856	-0.8825	0.4091
H	10.8636	-7.4315	3.6153	H	6.0415	6.6786	-4.4076	H	12.8906	-0.5363	-1.4973
H	13.0029	-1.8813	5.3656	H	7.4339	8.6934	-3.9823	H	14.1320	-2.4668	-2.4724
H	14.4099	-0.3331	4.0557	H	9.5910	8.4546	-2.7702	H	13.8210	-4.7245	-1.5009
H	14.1626	-0.2190	1.5830	H	13.0490	2.1578	-0.8695	H	12.9995	-6.0596	4.5314
H	12.4534	-1.6303	0.4542	H	14.1422	2.5615	-3.0663	H	11.7531	-5.2004	6.4798
H	10.5504	7.3193	-1.2347	H	12.8348	3.5976	-4.9092	H	10.0049	-3.4413	6.1090
H	9.0844	7.6882	-3.0051	H	10.4678	4.2102	-4.5628	H	9.6175	-2.5675	3.8748
H	10.8264	1.8211	-1.6038	H	-1.6017	-1.5867	0.3392	H	10.6927	3.3874	-0.0896
H	12.9650	2.0467	-0.4125	H	-2.4530	3.1284	1.6773	H	12.4307	3.6369	-0.1269
H	13.8600	4.3133	0.1189	H	-0.1467	2.2473	1.6726	H	12.2015	5.8853	-0.9931
H	12.5757	6.3136	-0.5608	H	-6.4181	3.4679	0.3882	H	11.2860	5.7081	0.4836
H	8.6658	7.3033	-5.2931	H	-8.6155	4.4589	0.8712	H	9.4206	3.0463	-4.7030
H	7.8949	5.8265	-7.1203	H	-10.0149	0.6377	2.2489	H	11.5065	2.1983	-5.7659
H	7.7714	3.3657	-6.7333	H	-7.8466	-0.3521	1.7139	H	13.6099	2.1142	-4.4493
H	8.4127	2.4338	-4.5527	H	-7.4258	0.2767	-1.4548	H	13.6309	2.8757	-2.0948
H	-1.6291	-1.5602	0.1855	H	-8.9761	-1.2499	-2.5678	H	10.7170	7.9786	-0.6021
H	-2.4015	3.1678	1.5255	H	-7.0955	-4.5861	-0.6212	H	8.8410	9.1924	-1.6359
H	-0.1108	2.2494	1.5175	H	-5.5029	-3.0492	0.4505	H	7.1209	7.9355	-2.9373
H	-6.3772	3.5784	0.2838	H	-10.6900	-2.7901	-0.5183	H	7.3329	5.4754	-3.1652
H	-8.5671	4.5749	0.7126	H	-10.3359	-6.2368	-1.9821	H	-1.6001	-1.5900	0.3105
H	-10.0311	0.7323	1.9918	H	-6.8008	-4.4579	-3.6438	H	-2.4544	3.1304	1.6263
H	-7.8629	-0.2473	1.5207	H	-6.0413	-6.6786	-4.4072	H	-0.1472	2.2490	1.6326
H	-7.3018	0.4027	-1.7041	H	-7.4341	-8.6933	-3.9827	H	-6.4713	3.4152	0.1135
H	-8.9275	-1.0767	-2.7900	H	-9.5914	-8.4545	-2.7712	H	-8.7147	4.3818	0.5347

H	-7.3352	-4.4288	-0.6483	H	-13.0490	-2.1584	-0.8685	H	-9.9737	0.6784	2.2822
H	-5.6645	-2.9407	0.4255	H	-14.1423	-2.5615	-3.0653	H	-7.7667	-0.2994	1.8229
H	-10.5513	-7.3191	-1.2341	H	-12.8350	-3.5969	-4.9086	H	-7.4681	0.2559	-1.4508
H	-9.0848	-7.6886	-3.0040	H	-10.4680	-4.2095	-4.5626	H	-9.0676	-1.2535	-2.4719
H	-10.8263	-1.8209	-1.6045	H	-9.2299	4.2642	3.8353	H	-7.1472	-4.5878	-0.5367
H	-12.9653	-2.0460	-0.4138	H	-12.7742	2.9747	3.6140	H	-5.5153	-3.0573	0.4428
H	-13.8609	-4.3123	0.1178	H	-11.3918	1.7698	-0.2662	H	-10.6927	-3.3867	-0.0901
H	-12.5767	-6.3130	-0.5611	H	-13.4835	0.5093	-0.6210	H	-12.4308	-3.6359	-0.1275
H	-8.6653	-7.3044	-5.2919	H	-15.2447	0.4838	1.1338	H	-12.2019	-5.8842	-0.9939
H	-7.8936	-5.8280	-7.1191	H	-14.8768	1.7304	3.2526	H	-11.2864	-5.7074	0.4829
H	-7.7697	-3.3672	-6.7326	H	-9.3742	6.5688	4.7071	H	-9.4206	-3.0454	-4.7035
H	-8.4116	-2.4347	-4.5524	H	-11.0653	8.1482	3.7989	H	-11.5063	-2.1969	-5.7663
H	-10.2654	5.4373	4.8603	H	-12.6008	7.3914	1.9967	H	-13.6097	-2.1126	-4.4497
H	-11.0719	3.3440	5.5209	H	-12.4359	5.0950	1.1052	H	-13.6308	-2.8743	-2.0952
H	-11.2510	4.7624	-0.5337					H	-10.7176	-7.9778	-0.6029
H	-11.4616	7.2387	-0.6302					H	-8.8418	-9.1918	-1.6368
H	-11.2375	8.5720	1.4578					H	-7.1217	-7.9351	-2.9382
H	-10.8635	7.4317	3.6150					H	-7.3332	-5.4749	-3.1659
H	-13.0029	1.8816	5.3654					H	-11.2083	5.7458	0.6362
H	-14.4100	0.3333	4.0555					H	-12.7729	6.1973	-0.0342
H	-14.1628	0.2192	1.5828					H	-13.8774	5.0812	1.9611
H	-12.4535	1.6306	0.4540					H	-12.9872	6.5450	2.3131
								H	-11.3839	0.8828	0.4072
								H	-12.8878	0.5375	-1.5002
								H	-14.1297	2.4683	-2.4743
								H	-13.8204	4.7252	-1.5006
								H	-13.0013	6.0561	4.5334
								H	-11.7540	5.1971	6.4812
								H	-10.0045	3.4395	6.1094
								H	-9.6167	2.5670	3.8749

Table SI4 presents the electronic properties of the **DQ** derivatives, including the $S_0 \rightarrow S_n$ (for $n = 1, \ldots, 20$) transition energies (E), as well as the corresponding wavelengths (λ) and oscillator strengths (f). As discussed in the main text, the theoretical results show an average deviation of ~0.45 eV for the $S_0 \rightarrow S_1$ transition, corresponding to a mean error of 16% relative to the experimental values. This overestimation is within the expected range for TD-CAM-B3LYP calculations with default parameters (μ = 0.33, α = 0.19, and β = 0.46)[28], whose fine-tuning could improve agreement for **DQs**, as demonstrated by Okuno *et al.*[29] However, such a procedure requires dedicated parameterization, which is beyond the scope of the present study[28]. Therefore, to allow a more direct comparison, a uniform shift was applied to the theoretical transition energies to align them with the experimental values. The applied shifts were 0.47 eV, 0.42 eV, and 0.46 eV for **DQ-IMD**, **DQ-DPA**, and **DQ-DDA,** respectively, as indicated in **Fig. 2a-c** and **Table SI4**. The transition energy and wavelength values in **Fig. 2a-c** and **Table SI4** already include the shift.

Table SI4 – Electronic properties of **DQ** derivatives, including transition energies (E), wavelengths (λ), and oscillator strengths (f) obtained from IEFPCM-TD-CAM-B3LYP/6-311++G(d,p) calculations. E and λ were red-shifted by 0.47, 0.42, and 0.46 eV, respectively.

IEFPCM-TD-CAM-B3LYP/6-311++G(d,p)			
	**DQ-IMD**	**DQ-DPA**	**DQ-DDA**

Electronic state	$E\ (eV)$	$\lambda\ (nm)$	f	$E\ (eV)$	$\lambda\ (nm)$	f	$E\ (eV)$	$\lambda\ (nm)$	f
	Redshifted by 0.47 eV			Redshifted by 0.42 eV			Redshifted by 0.46 eV		
S_0-S_1	2.857	434	2.13	2.799	443	2.39	2.924	424	1.90
S_0-S_2	2.964	418	0.05	2.934	423	0.02	3.016	411	0.05
S_0-S_3	2.964	418	0.04	3.066	404	0.61	3.264	380	0.08
S_0-S_4	3.011	412	0.00	3.081	402	0.25	3.278	378	0.00
S_0-S_5	3.238	383	0.01	3.268	379	0.01	3.321	373	0.33
S_0-S_6	3.246	382	0.00	3.270	379	0.01	3.401	365	0.07
S_0-S_7	3.268	379	0.00	3.402	364	0.06	3.426	362	0.05
S_0-S_8	3.268	379	0.03	3.404	364	0.41	3.429	362	0.11
S_0-S_9	3.360	369	0.32	3.429	362	0.01	3.502	354	1.34
S_0-S_{10}	3.380	367	0.13	3.446	360	0.42	3.560	348	0.04
S_0-S_{11}	3.556	349	0.03	3.480	356	0.03	3.710	334	0.00
S_0-S_{12}	3.556	349	0.12	3.482	356	0.07	3.711	334	0.06
S_0-S_{13}	3.583	346	0.28	3.503	354	0.06	3.731	332	0.08
S_0-S_{14}	3.590	345	0.01	3.510	353	0.00	3.754	330	0.00
S_0-S_{15}	3.642	340	0.05	3.519	352	0.33	3.779	328	0.05
S_0-S_{16}	3.643	340	0.90	3.617	343	0.02	3.780	328	0.09
S_0-S_{17}	3.645	340	0.54	3.628	342	0.79	3.833	323	0.28
S_0-S_{18}	3.674	337	0.03	3.631	341	0.02	3.838	323	0.00
S_0-S_{19}	3.721	333	0.04	3.638	341	0.39	3.873	320	0.30
S_0-S_{20}	3.722	333	0.00	3.639	341	0.02	3.902	318	0.00

Table SI5 – Electronic parameters of the **DQ** derivatives, including the coordinates of the centroids of positive and negative charge (x, y, and z: R_+ and R_-), the effective charge displacement length (D_{CT}), and the transferred charge (q_{CT}), determined by QCC at the IEFPCM-TD-CAM-B3LYP/6-311++G(d,p) level.

Sample	R_+			R_-			D_{CT} (Å)	q_{CT} $(\|e^-\|)$
	x (Å)	y (Å)	z (Å)	x (Å)	y (Å)	z (Å)		
**DQ-IMD**	-0.012	0.003	0.713	-0.006	0.001	1.089	0.38	0.72
**DQ-DPA**	0.002	0.0	0.706	0.004	0.001	0.553	0.15	0.73
**DQ-DDA**	0.0	0.0	0.263	0.0	0.001	-0.701	0.96	0.76

We also applied the procedure described above to the 2PA transition energies obtained via QRF-CAM-B3LYP/6-311+G(d). In this case, we used uniform energy redshifts of 0.29, 0.27, and 0.29 eV for **DQ-IMD**, **DQ-DPA**, and **DQ-DDA**, respectively. The values presented in **Table SI6** already correspond to the redshifted energies. It is worth noting that, since these calculations were performed in the gas phase, this additional factor may contribute to the differences observed about the experimental results.

Although QCCs exhibit deviations from experimental results, they provide useful insights that aid in interpreting the data. For instance, the comparison between **Tables SI6** and **SI3** reveals that transitions that are strongly one-photon allowed tend to be weakly two-photon allowed, and vice versa, except for slight variations. This behavior is consistent with the discussion in the main text, where we attribute this feature to the quasi-centrosymmetric quadrupolar nature of the molecules. Nevertheless, it is essential to note that we cannot entirely rule out the possibility that certain states are allowed in both 1PA and 2PA processes, especially considering the structural

flexibility of **DQ** derivatives, which may promote symmetry breaking already in the ground state (although this behavior was not observed).

Table SI6 – Theoretical two-photon absorption properties of **DQ** derivatives obtained by QRF-CAM-B3LYP/6-311++G(d,p) calculations in gas phase; including 2PA transition energies (E_{2PA}), 2PA wavelengths (λ_{2PA}), 2PA probability, and 2PA cross-section (σ_{2PA}). E_{2PA} and λ_{2PA} were redshifted by 0.29, 0.27, and 0.29 eV, respectively.

QRF-CAM-B3LYP/6-311++G(d,p)				
**DQ-IMD**				
	Redshifted by 0.29 eV			
**Electronic state**	E_{2PA} (eV)	λ_{2PA} **(nm)**	**2PA prob. (** $\times 10^3$ $a.u.$**)**	σ_{theor}^{2PA} (GM)
S_0-S_1	1.428	868	0.37	0.6
S_0-S_2	1.439	862	0.02	0.0
S_0-S_3	1.439	862	0.02	0.0
S_0-S_4	1.503	825	32.50	63.8
S_0-S_5	1.547	801	0.03	0.1
S_0-S_6	1.547	801	0.02	0.1
S_0-S_7	1.560	795	0.00	0.0
S_0-S_8	1.564	793	0.01	0.0
S_0-S_9	1.636	758	0.61	1.5
S_0-S_{10}	1.652	751	57.60	142.7
**DQ-DPA**				
	Redshifted by 0.27 eV			
**Electronic state**	E_{2PA} (eV)	λ_{2PA} **(nm)**	**2PA prob. (** $\times 10^3$ $a.u.$**)**	σ_{theor}^{2PA} (GM)
S_0-S_1	1.399	886	0.18	0.3
S_0-S_2	1.473	842	69.90	128.4
S_0-S_3	1.514	819	6.83	13.4
S_0-S_4	1.517	817	21.30	42.1
S_0-S_5	1.575	787	0.23	0.5
S_0-S_6	1.575	787	1.05	2.3
S_0-S_7	1.665	745	1.14	2.8
S_0-S_8	1.671	742	5.03	12.6
S_0-S_9	1.675	740	68.40	171.9
S_0-S_{10}	1.681	737	1.99	5.0
**DQ-DDA**				
	Redshifted by 0.29 eV			
**Electronic state**	E_{2PA} (eV)	λ_{2PA} **(nm)**	**2PA prob. (** $\times 10^3$ $a.u.$**)**	σ_{theor}^{2PA} (GM)
S_0-S_1	1.462	848	1.54	2.8
S_0-S_2	1.511	821	29	56.3
S_0-S_3	1.575	787	0.188	0.4
S_0-S_4	1.579	785	0.528	1.1
S_0-S_5	1.619	766	1.62	3.7
S_0-S_6	1.636	758	2.18	5.1
S_0-S_7	1.656	749	0.582	1.4
S_0-S_8	1.672	741	11.9	29.6
S_0-S_9	1.715	723	0.296	0.8
S_0-S_{10}	1.747	710	27.7	76.9

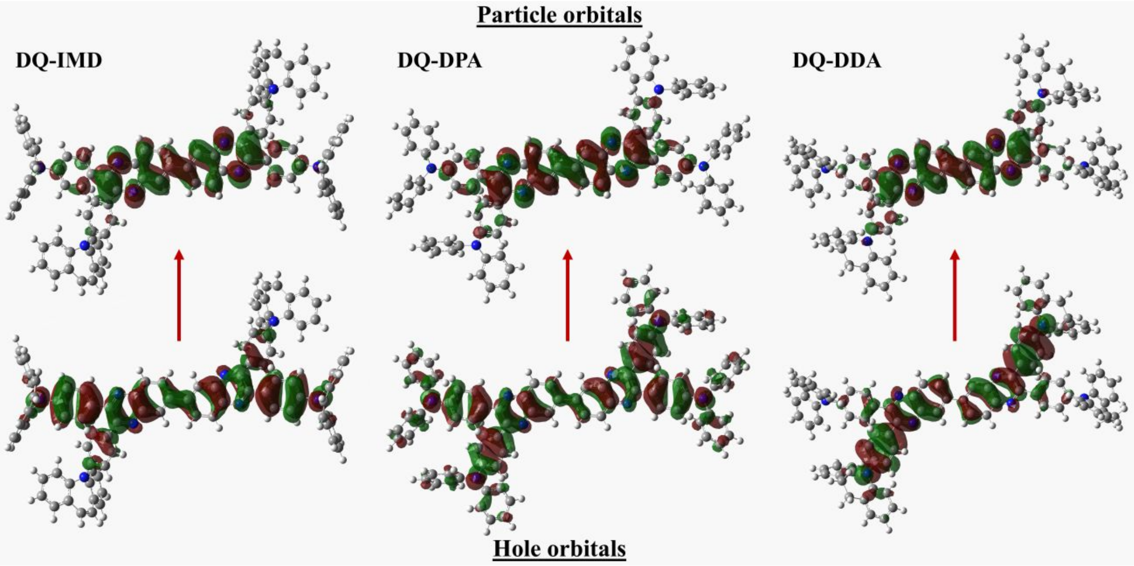

Figure SI6 – Natural transition orbitals corresponding to the $S_0 \rightarrow S_4$ transition for **DQ-IMD** and to the $S_0 \rightarrow S_2$ one for **DQ-DPA** and **DQ-DDA**, obtained from quantum chemical calculations at the PCM-TD-CAM-B3LYP/6-311++G(d,p) level (isocontour value: 0.015 a.u.). These are the lowest-energy transitions strongly allowed ($2PA\ prob. > 10^4\ a.u.$) by two photons absorption.

SI6 Multiphoton polymerization

To evaluate the performance of the **DQ** derivatives as two-photon photoinitiators (PIs), we prepared a 1:1 (w/w) mixture of two acrylic resins: *ethoxylated(6)trimethylolpropane triacrylate* (SR499 – Sartomer) and *tris(2-hydroxyethyl)isocyanurate triacrylate* (SR368 – Sartomer) (see **Fig. SI7**). This combination provides good mechanical stability and flexibility, while minimizing intrinsic shrinkage during photopolymerization[30]. We incorporated different concentrations of the **DQs** (0.05 ± 0.01 – 0.22 ± 0.01 wt%), previously dissolved in DCM, into the viscous resin mixture. After evaporating the solvent, we deposited a small drop of the mixture onto a glass substrate with micrometric spacers and covered it with a coverslip. We performed microfabrication via two-photon polymerization (2PP) and subsequently washed the sample to remove excess uncured resin by immersing it in ethanol at approximately 75 °C for 15 minutes. We repeated this procedure three times.

We employed a homemade setup to manufacture the microstructures by 2PP. Briefly, the setup consists of an amplified femtosecond erbium laser system (FemtoFiber Scientific model, Toptica Photonics, *Inc.*), with the following specifications: a 200-fs pulse duration at 775 nm (the second harmonic of 1550 nm) and a repetition rate of 80 MHz (0.75 nJ/pulse). We controlled the laser beam power using a half-wave plate combined with a polarizer. We then focused the beam onto the sample through a 62× objective lens (NA = 0.80), which was positioned at an x-y-z translation

stage to define the geometry of the fabricated microstructures. We set the stage translation speed to 20 µm/s and adjust the incident laser power on the sample from 3.19 ± 0.28 mW to 7.44 ± 0.51 mW (0.040 ± 0.006 – 0.093 ± 0.003 nJ).

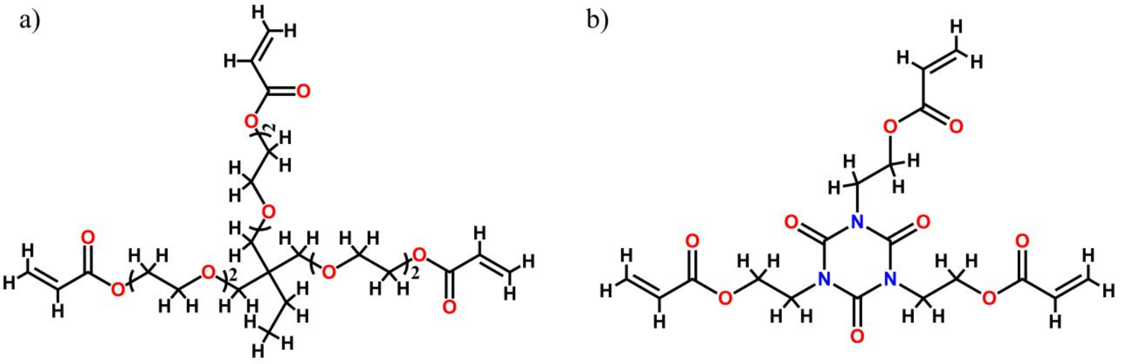

SR499 – *ethoxylated(6)trimethylolpropane triacrylate* **SR368** – *tris(2-hydroxyethyl)isocyanurate triacrylate*

Figure SI7 – Chemical structures of the monomers: a) SR499-Sartomer and b) SR368-Sartomer.

SI7 NMR spectrum of compounds

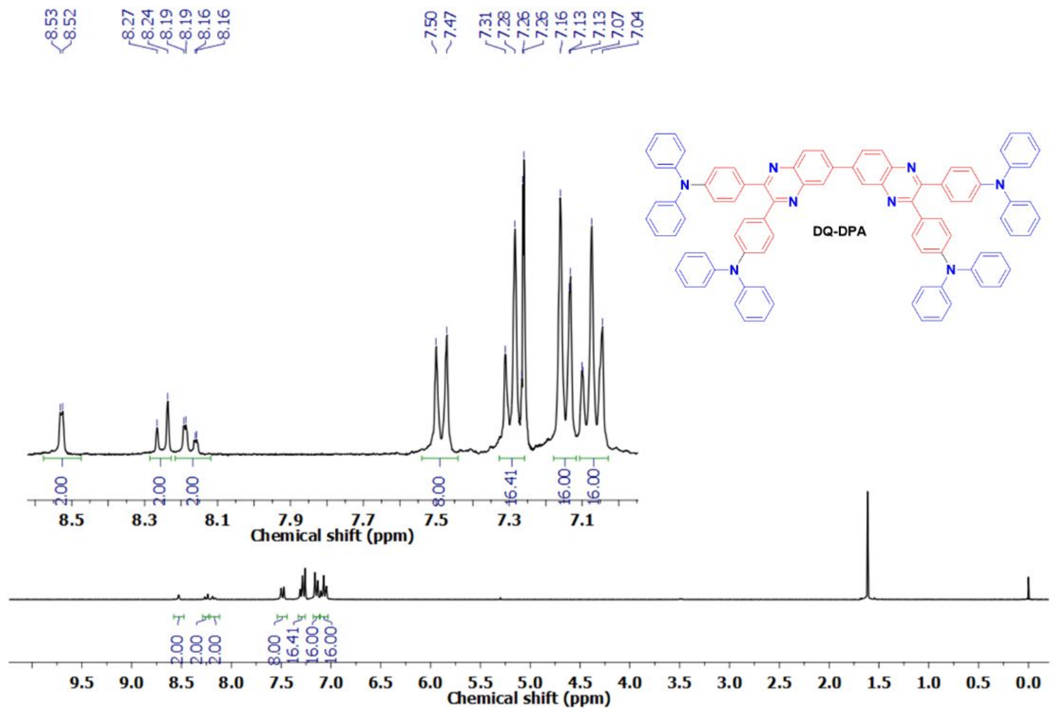

*^1H NMR (300 MHz) in $CDCl_3$ of the compound **DQ-DPA.***

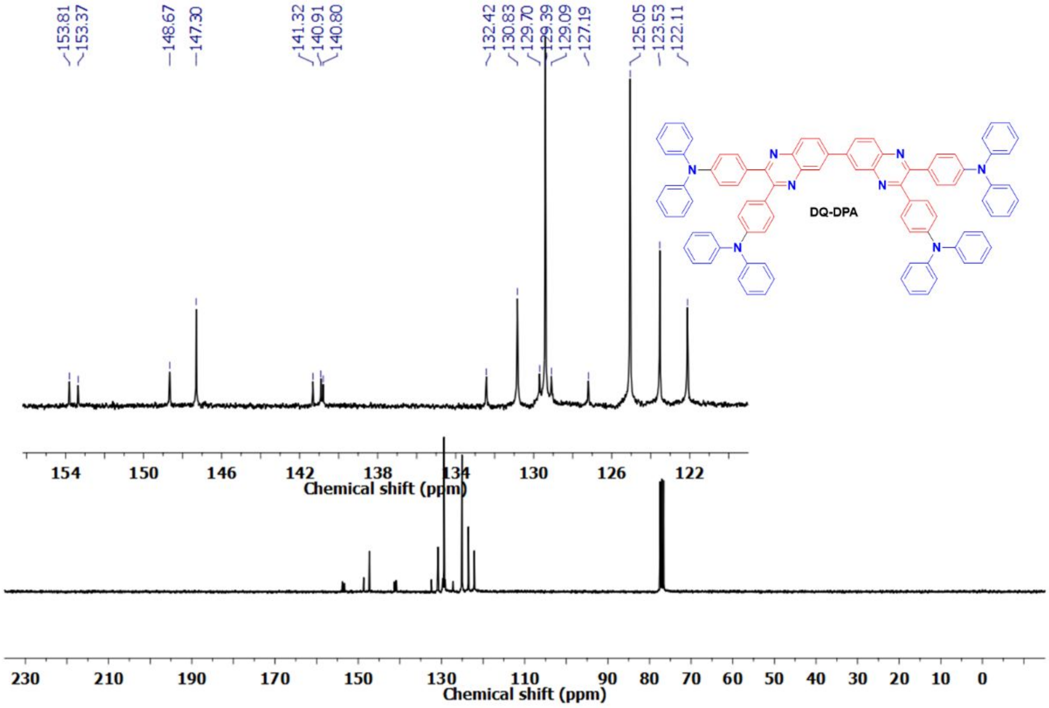

^{13}C NMR (75 MHz) in $CDCl_3$ of the compound ***DQ-DPA.***

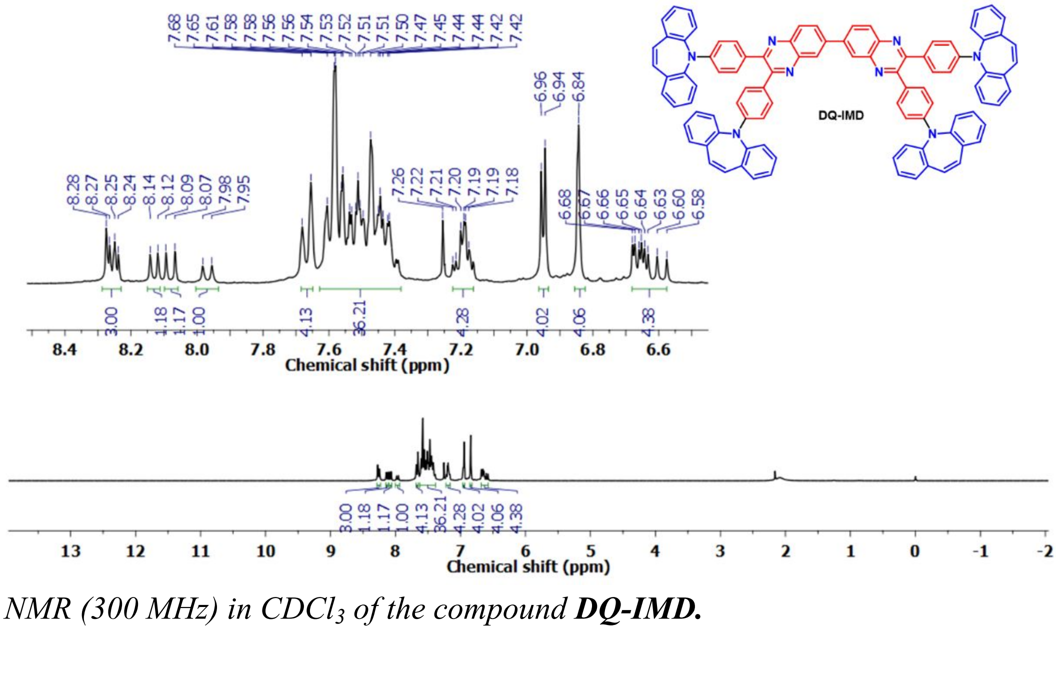

^{1}H NMR (300 MHz) in $CDCl_3$ of the compound ***DQ-IMD.***

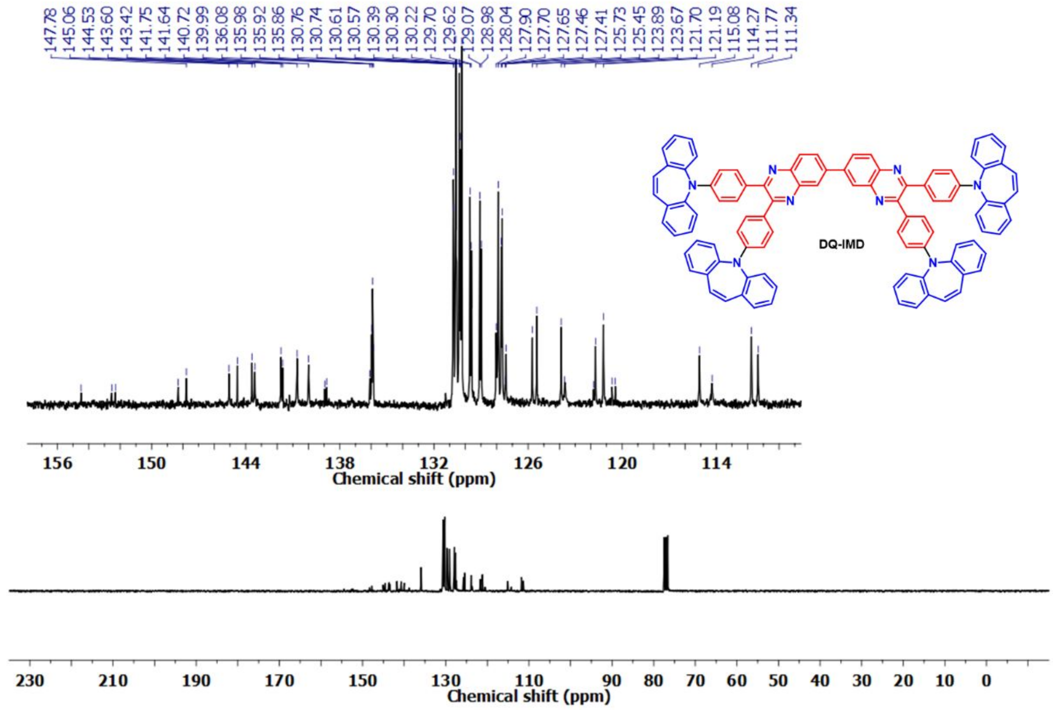

*^{13}C NMR (75 MHz) in $CDCl_3$ of the compound **DQ-IMD**.*

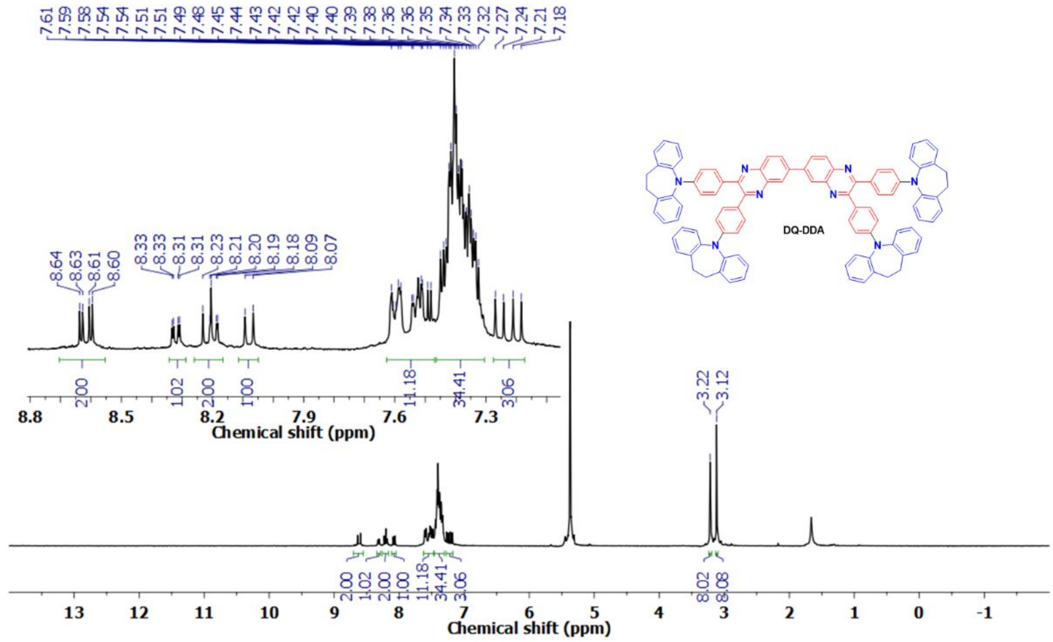

*^{1}H NMR (300 MHz) in CD_2Cl_2 of the compound **DQ-DDA**.*

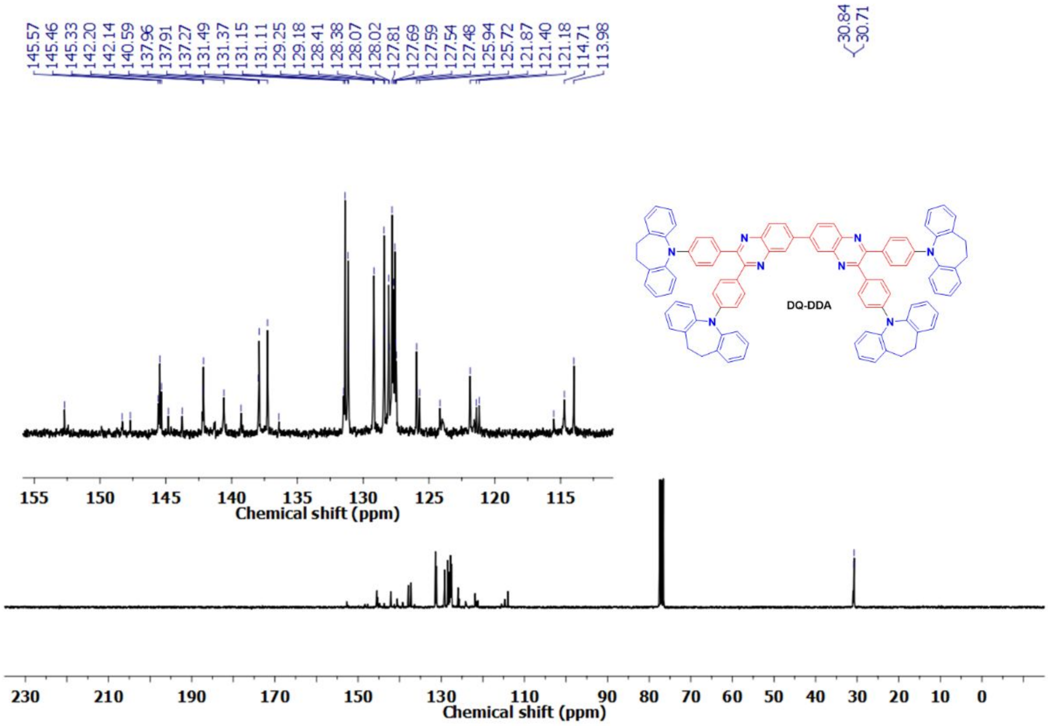

^{13}C NMR (75 MHz) in $CDCl_3$ of the compound **DQ-DDA.**

SI8 References

1. Brouwer AM. Standards for photoluminescence quantum yield measurements in solution (IUPAC technical report). *Pure and Applied Chemistry*. 2011;83(12):2213-2228. doi:10.1351/PAC-REP-10-09-31

2. Würth C, Grabolle M, Pauli J, Spieles M, Resch-Genger U. Relative and absolute determination of fluorescence quantum yields of transparent samples. *Nat Protoc*. 2013;8(8):1535-1550. doi:10.1038/nprot.2013.087

3. Rohwer LS, Martin JE. Measuring the absolute quantum efficiency of luminescent materials. *J Lumin*. 2005;115(3-4):77-90. doi:10.1016/j.jlumin.2005.01.013

4. Joseph R. Lakowicz. Fluorescence Anisotropy. In: Lakowicz JR, ed. *Principles of Fluorescence Spectroscopy*. Springer US; 2006:353-382. doi:10.1007/978-0-387-46312-4_10

5. Yan L, Chen X, He Q, et al. Localized Emitting State and Energy Transfer Properties of Quadrupolar Chromophores and (Multi)Branched Derivatives. *J Phys Chem A*. 2012;116(34):8693-8705. doi:10.1021/jp305407s

6. Reichardt C, Welton T. Solvents and Solvent Effects in Organic Chemistry: Fourth Edition. *Solvents and Solvent Effects in Organic Chemistry: Fourth Edition*. Published online November 26, 2010. doi:10.1002/9783527632220

7. Lide DR. CRC Handbook of Chemistry and Physics, Internet Version 2005. *CRC Press, Taylor and Francis Boca Raton FL*. Published online 2005:2660.

8. Zucolotto Cocca LH, Valverde JVP, le Bescont J, et al. Photophysical properties of 3-arylthioimidazo[1,2-a]pyridine derivatives: The role of peripheral electron-donating and electron-withdrawing groups in the advance of organic materials engineering. *J Mol Struct*. 2024;1300:137221. doi:10.1016/j.molstruc.2023.137221

9. Zucolotto Cocca LH, Pelosi AG, Valverde JVP, et al. 3-arylthioimidazo[1,2-a]pyridine derivatives: A theoretical and experimental study of its photophysical properties. *J Photochem Photobiol A Chem*. 2023;440:114675. doi:10.1016/j.jphotochem.2023.114675

10. Toptygin D. Effects of the Solvent Refractive Index and Its Dispersion on the Radiative Decay Rate and Extinction Coefficient of a Fluorescent Solute. *J Fluoresc*. 2003;13(3):201-219. doi:10.1023/A:1025033731377

11. Ivanov AI, Dereka B, Vauthey E. A simple model of solvent-induced symmetry-breaking charge transfer in excited quadrupolar molecules. *Journal of Chemical Physics*. 2017;146(16). doi:10.1063/1.4982067

12. Dereka B, Rosspeintner A, Stężycki R, Ruckebusch C, Gryko DT, Vauthey E. Excited-State Symmetry Breaking in a Quadrupolar Molecule Visualized in Time and Space. *Journal of Physical Chemistry Letters*. 2017;8(24):6029-6034. doi:10.1021/acs.jpclett.7b02944

13. Nazarov AE, Ivanov AI, Vauthey E. Modeling Infrared Spectral Dynamics upon Symmetry Breaking of a Photo-Excited Quadrupolar Dye. *Journal of Physical Chemistry C*. 2020;124(4):2357-2369. doi:10.1021/acs.jpcc.9b10565

14. Makarov NS, Drobizhev M, Rebane A. Two-photon absorption standards in the 550-1600 nm excitation wavelength range. *Opt Express*. 2008;16(6):4029. doi:10.1364/OE.16.004029

15. Xu C, Webb WW. *Measurement of Two-Photon Excitation Cross Sections of Molecular Fluorophores with Data from 690 to 1050 Nm*. Vol 13.; 1996.

16. de Reguardati S, Pahapill J, Mikhailov A, Stepanenko Y, Rebane A. High-accuracy reference standards for two-photon absorption in the 680–1050 nm wavelength range. *Opt Express*. 2016;24(8):9053. doi:10.1364/oe.24.009053

17. Valverde JVP, Romero AL do S, Cunha R, et al. The ESIPT-suppressed 2-(2′-hydroxyphenyl)benzoxazole derivative as a new photoinitiator for multiphoton polymerization. *J Mater Chem C Mater*. Published online February 22, 2025. doi:10.1039/d4tc05477f

18. M. J. Frisch, G. W. Trucks, H. B. Schlegel, et al. Gaussian 09, Revision A.02. Published online 2009.

19. Becke AD. Density-functional thermochemistry. III. The role of exact exchange. *J Chem Phys*. 1993;98(7):5648-5652. doi:10.1063/1.464913

20. Woon DE, Dunning TH. Gaussian basis sets for use in correlated molecular calculations. V. Core-valence basis sets for boron through neon. *J Chem Phys*. 1995;103(11):4572-4585. doi:10.1063/1.470645

21. Yanai T, Tew DP, Handy NC. A new hybrid exchange-correlation functional using the Coulomb-attenuating method (CAM-B3LYP). *Chem Phys Lett*. 2004;393(1-3):51-57. doi:10.1016/j.cplett.2004.06.011

22. Martin RL. Natural transition orbitals. *Journal of Chemical Physics*. 2003;118(11):4775-4777. doi:10.1063/1.1558471

23. Le Bahers T, Adamo C, Ciofini I. A qualitative index of spatial extent in charge-transfer excitations. *J Chem Theory Comput*. 2011;7(8):2498-2506. doi:10.1021/ct200308m

24. Tomasi J, Mennucci B, Cancès E. The IEF version of the PCM solvation method: an overview of a new method addressed to study molecular solutes at the QM ab initio level. *Journal of Molecular Structure: THEOCHEM*. 1999;464(1-3):211-226. doi:10.1016/S0166-1280(98)00553-3

25. Cancès E, Mennucci B, Tomasi J. A new integral equation formalism for the polarizable continuum model: Theoretical background and applications to Isotropic and anisotropic dielectrics. *Journal of Chemical Physics*. 1997;107(8):3032-3041. doi:10.1063/1.474659

26. Sałek P, Vahtras O, Guo J, Luo Y, Helgaker T, Ågren H. Calculations of two-photon absorption cross sections by means of density-functional theory. *Chem Phys Lett*. 2003;374(5-6):446-452. doi:10.1016/S0009-2614(03)00681-X

27. Aidas K, Angeli C, Bak KL, et al. The Dalton quantum chemistry program system. *Wiley Interdiscip Rev Comput Mol Sci*. 2014;4(3):269-284. doi:10.1002/WCMS.1172

28. Vivas MG, Silva DL, Malinge J, et al. Molecular Structure – Optical Property Relationships for a Series of Non-Centrosymmetric Two-photon Absorbing Push-Pull Triarylamine Molecules. *Sci Rep*. 2014;4(1):4447. doi:10.1038/srep04447

29. Okuno K, Shigeta Y, Kishi R, Miyasaka H, Nakano M. Tuned CAM-B3LYP functional in the time-dependent density functional theory scheme for excitation energies and properties of diarylethene derivatives. *J Photochem Photobiol A Chem*. 2012;235:29-34. doi:10.1016/J.JPHOTOCHEM.2012.03.003

30. Baldacchini T, LaFratta CN, Farrer RA, et al. Acrylic-based resin with favorable properties for three-dimensional two-photon polymerization. *J Appl Phys*. 2004;95(11 I):6072-6076. doi:10.1063/1.1728296
